# The genetic component of human longevity: New insights from the analysis of pathway‐based SNP‐SNP interactions

**DOI:** 10.1111/acel.12755

**Published:** 2018-03-25

**Authors:** Serena Dato, Mette Soerensen, Francesco De Rango, Giuseppina Rose, Kaare Christensen, Lene Christiansen, Giuseppe Passarino

**Affiliations:** ^1^ Department of Biology, Ecology and Earth Sciences University of Calabria Rende Italy; ^2^ The Danish Aging Research Center, Epidemiology, Biostatistics and Biodemography Institute of Public Health University of Southern Denmark Odense C Denmark; ^3^ Department of Clinical Genetics Odense University Hospital Odense C Denmark

**Keywords:** aging, epistasis, genetic component of human longevity, pathway‐based analysis, SNP, synergic interaction

## Abstract

In human longevity studies, single nucleotide polymorphism (SNP) analysis identified a large number of genetic variants with small effects, yet not easily replicable in different populations. New insights may come from the combined analysis of different SNPs, especially when grouped by metabolic pathway. We applied this approach to study the joint effect on longevity of SNPs belonging to three candidate pathways, the insulin/insulin‐like growth factor signalling (IIS), DNA repair and pro/antioxidant. We analysed data from 1,058 tagging SNPs in 140 genes, collected in 1825 subjects (1,089 unrelated nonagenarians from the Danish 1905 Birth Cohort Study and 736 Danish controls aged 46–55 years) for evaluating synergic interactions by SNPsyn. Synergies were further tested by the multidimensional reduction (MDR) approach, both intra‐ and interpathways. The best combinations (FDR<0.0001) resulted those encompassing *IGF1R‐*rs12437963 and *PTPN1‐*rs6067484, *TP53‐*rs2078486 and *ERCC2‐*rs50871, *TXNRD1‐*rs17202060 and *TP53‐*rs2078486, the latter two supporting a central role of *TP53* in mediating the concerted activation of the DNA repair and pro‐antioxidant pathways in human longevity. Results were consistently replicated with both approaches, as well as a significant effect on longevity was found for the *GHSR* gene, which also interacts with partners belonging to both IIS and DNA repair pathways (*PAPPA*,*PTPN1*,*PARK7, MRE11A*). The combination *GHSR‐MREA11*, positively associated with longevity by MDR, was further found influencing longitudinal survival in nonagenarian females (*p *= .026). Results here presented highlight the validity of SNP‐SNP interactions analyses for investigating the genetics of human longevity, confirming previously identified markers but also pointing to novel genes as central nodes of additional networks involved in human longevity.

## 1 INTRODUCTION

Association studies can identify the association of individual gene variants to a given phenotype. Nevertheless, such analysis is unable to explain the biological complexity of several diseases and complex phenotypes such as human aging and longevity.

Recent advances in DNA technology and association studies have uncovered a very large number of common susceptibility variants for complex traits; however, the translation into practice of their role is complicated by the evidence that such variants operate together to influence the final phenotype. The major challenge is represented by the relatively small genetic contribution to the variation in phenotype, which in human lifespan was estimated to be approximately 25% (Herskind et al., 1996) determined by many, mostly still uncharacterized, genes (Deelen et al., 2013), possibly belonging to conserved pathways (Johnson, Dong, Vijg & Suh, 2015). Single‐SNP analyses may miss such a complexity, primarily because if a genetic factor operates through a mechanism involving multiple genes, and is also affected by environmental factors, the single investigation may not examine statistical interactions between loci that are informative about the biological and biochemical pathways underpinning the phenotype. On the contrary, SNP interactions may carry more information about the phenotype than those observed from individual SNPs alone (Gerke, Lorenz & Cohen, 2009; Su et al., 2015). Assessing SNP‐SNP interactions at the gene level co‐occurring within a specific phenotype and not due to linkage disequilibrium (LD) can overcome this problem, possibly finding specific subprocesses more strongly associated with the phenotype than single‐SNP analysis (Cordell, 2009). However, the search of all possible SNP‐SNP interactions is challenging from an analytical point of view, because of the number of pairwise statistical tests to be performed. Only recently, significant advances in statistical approaches make it possible to analyse multiple SNPs in large genetic data sets, considering the main pathway to which the gene belongs and possible covariates, as required in the analysis of complex traits (Curk, Rot & Zupan, 2011).

Several SNPs in genes belonging to distinct pathways have been associated with the longevity phenotype (Soerensen et al., 2012; Dato et al., 2013 and references therein, Rose et al., 2015; Crocco, Montesanto, Passarino & Rose, 2016; De Luca, Crocco, De Rango, Passarino & Rose, 2016). GWAS of human longevity in worldwide samples (North America, Europe and very recently China) generally failed to give new insights into genetic determinants of human longevity: only the *TOMM40/APOE/APOC1* locus, associated with longevity, was replicated in different populations (Deelen et al., 2014; Lin et al., 2016; Newman et al., 2010; Sebastiani et al., 2012), while rs2149954 on 5q33.3 (Deelen et al., 2014; Zeng et al., 2016) and the *FOXO3A* locus (Broer et al., 2015 and references therein) are the other signals showing population‐specific associations. Some authors attempted to analyse epistatic intragenic (Tan, Soerensen, Kruse, Christensen & Christiansen, 2013; Zeng et al., 2010) or intergenic effects (Napolioni, Giannì, Carpi, Predazzi & Lucarini, 2011a; Napolioni et al., 2011b), however comprising a limited number of genetic variants. Recently, the analysis of the joint effect of a SNP set grouped by pathway or gene region (pathway/group‐based analysis) on human longevity was carried out by Deelen and co‐authors (Deelen et al., 2013), who analysed GWAS data of 1,021 SNPs in 68 genes from the insulin‐Igf1 signalling (IIS) pathway and 88 SNPs in 13 telomere maintenance (TM) pathway genes. The comparison of genotype data between 403 unrelated nonagenarians from the Leiden Longevity Study and 1,670 younger population controls confirmed the role of genetic variation in IIS and TM pathways in the predisposition to longevity. However, while IIS had many genes associated with the trait, the association of TM with survival was mainly determined by the *POT1* gene. Similarly, we previously performed a pathway analysis of 592 SNPs in the DNA repair pathway in the same in the same study population as investigated in the present study indicating association of subprocesses of this pathway in human longevity (Debrabant et al., 2014). Here, we wanted to explore synergies inside a large SNP data set, by applying statistical methodologies allowing us to test the interaction both inside and among SNP variants belonging to three main candidate pathways of human longevity such as IIS, DNA damage signalling and repair, and pro/antioxidant response. To this aim, we analysed 1,058 SNPs from 140 genes in 1,825 subjects (1,089 cases aged 92–93, 736 controls aged 46–55) of Danish origin, previously analysed for single‐SNP associations with longevity (Soerensen et al., 2012).

## 2 RESULTS

In this work, we used a combination of different approaches for understanding the relationships between SNP variants in the predisposition to become long‐lived. First, we computed case–control based SNP‐SNP interaction without pathway constraints using the whole data set, and we plotted interaction networks from significant SNP combinations. Then, we performed pathway‐based case–control analysis, looking for epistatic interactions inside original assigned pathways and among different pathways by applying a MDR approach. Finally, we analysed those pairs of SNPs significantly enriched in cases respect to controls for their influence on survival in the oldest old.

### 2.1 SNPsyn analysis in the entire data set

**Figure 1:**
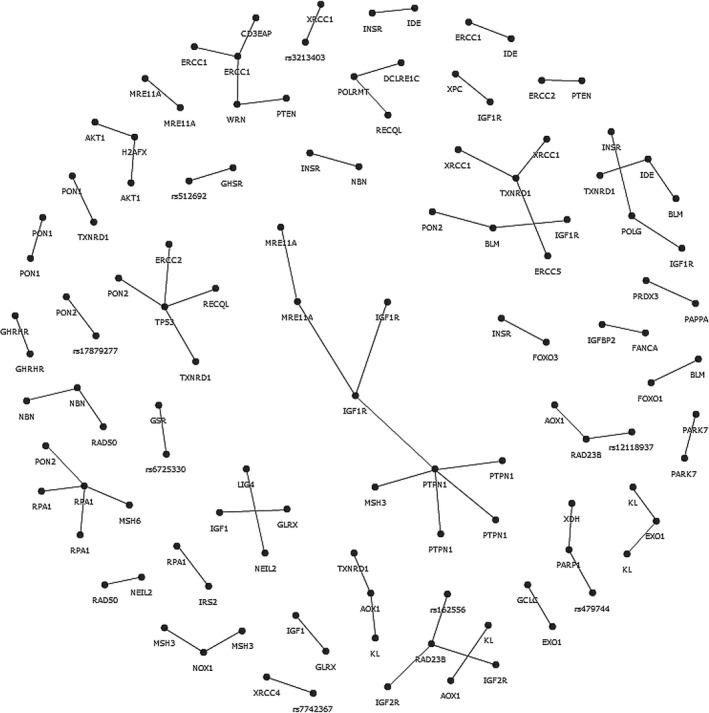
Interaction network obtained by the analysis of SNP‐SNP interactions by SNPsyn in the whole data set, comprising the all three analysed pathways (IIS, DNA repair and pro‐antioxidant). Each dot represents a SNP, indicated by the corresponding gene, when univocally assigned or by dbSNP notation, when present in a regulatory region, and not assigned by the SNPsyn output

By testing all possible combinations among the 1,058 available variants, assuming a significance level *p *< .0001 and correcting for multiple comparisons (FDR <0.005), we found a set of 73 best‐interacting SNP pairs (Table S1). Figure 1 shows the interaction network, composed by 33 intrapathway network (11 IIS; 17 DNA repair; 5 pro‐antioxidant) and 40 interpathway network (22 IIS‐DNA repair; 11 pro‐antioxidant‐DNA repair; 7 pro‐antioxidant‐IIS). Sixteen of 73 SNP‐SNP interactions were inside the same gene: these interactions could be due to noncasual association among markers (LD), however modest (*r* ^2^ < 0.8), as indicated by the positive values of synergy (Syn) among variants (all interactions reported significant showed a Syn >0). In any case, the single contributions (I1 and I2) of SNP1 and SNP2 to the synergic interaction can give information about the more influencing SNP of the pairs and thus help in prioritizing the SNP in future analysis, so that no one SNP was *a priori* removed (Tables 1 and S1).

**Table 1:** List of top‐ranked 22 SNP‐SNP interactions, sorted by FDR <= 0.001, organized according to the synergy value, as resulting from SNPsyn analysis on the whole genotypic data set [1825 samples (1089 long‐lived, 736 younger controls), 1059 SNPs]. SNP‐SNP interactions inside the same gene captured by the algorithm could be due to a modest LD between markers, below the *r* ^2^ = 0.8 used as cutoff. Syn: synergy value, FDR: false discovery rate; I: information, I1 and I2: contribution of SNP1 and SNP2 to the synergic interaction, respectively

Syn	I	*p*‐value	FDR	Snp 1	Pathway	Chr 1	Snp 2	Y37	Chr 2	I 1	I 2
0.0205	0.0325	<.0001	<0.0001	GHSR (rs512692)	IIS	3	GHSR (rs572169)	IIS	3	0.0006	0.0115
0.0199	0.0246	<.0001	<0.0001	ERCC1 (rs3212961)	DNA repair	19	ERCC1 (rs762562)	DNA repair	19	0.0034	0.0013
0.0181	0.0297	<.0001	<0.0001	GHRHR (rs2267723)	IIS	7	GHRHR (rs4988505)	IIS	7	0.0112	0.0004
0.0159	0.0214	<.0001	<0.0001	PTPN1 (rs2038526)	IIS	20	PTPN1 (rs6067484)	IIS	20	0.0001	0.0054
0.0143	0.0178	<.0001	<0.0001	ERCC1 (rs3212961)	DNA repair	19	ERCC1 (rs3212964)	DNA repair	19	0.0034	0.0001
0.0140	0.0182	<.0001	<0.0001	NBN (rs12680687)	DNA repair	8	NBN (rs2735385)	DNA repair	8	0.0041	0.0001
0.0126	0.0182	<.0001	<0.0001	PTPN1 (rs2426164)	IIS	20	PTPN1 (rs6067484)	IIS	20	0.0002	0.0054
0.0125	0.0165	<.0001	<0.0001	XRCC1 (rs1799782)	DNA repair	19	XRCC1 (rs3213403)	DNA repair	19	0.0005	0.0035
0.0120	0.0189	<.0001	<0.0001	TXNRD1 (rs17202060)	Pro/Anti oxidant	12	TP53 (rs2078486)	DNA repair	17	0.0007	0.0062
0.0112	0.0196	<.0001	<0.0001	IGF1R (rs12437963)	IIS	15	PTPN1 (rs6067484)	IIS	20	0.0030	0.0054
0.0112	0.0186	<.0001	<0.0001	TP53 (rs2078486)	DNA repair	17	ERCC2 (rs50871)	DNA repair	19	0.0062	0.0012
0.0102	0.0165	<.0001	0.0006	PTPN1 (rs6063534)	IIS	20	PTPN1 (rs6067484)	IIS	20	0.0008	0.0054
0.0098	0.0157	<.0001	0.0006	RPA1 (rs11656253)	DNA repair	17	RPA1 (rs17292175)	DNA repair	17	0.0058	9,61E‐01
0.0097	0.0155	<.0001	0.0006	MRE11A (rs512150)	DNA repair	11	MRE11A (rs592068)	DNA repair	11	0.0041	0.0017
0.0091	0.0156	<.0001	0.0006	AOX1 (rs2465661)	Pro/Anti oxidant	2	RAD23B (rs11573709)	DNA repair	9	0.0024	0.0041
0.0087	0.0161	<.0001	0.0009	WRN (rs11574218)	DNA repair	8	ERCC1 (rs3212961)	DNA repair	19	0.0040	0.0034
0.0084	0.0158	<.0001	0.0009	AOX1 (rs2256977)	Pro/Anti oxidant	2	KL (rs2283368)	IIS	13	0.0039	0.0035
0.0081	0.0154	<.0001	0.0009	PARP1 (rs1136410)	DNA repair	1	FOXO3A (rs479744)	IIS	6	0.0048	0.0024
0.0127	0.0147	<.0001	0.0012	PRDX3 (rs1553850)	Pro/Anti oxidant	10	PAPPA (rs449807)	IIS	9	0.0010	0.0011
0.0122	0.0139	<.0001	0.0012	MRE11A (rs10831227)	DNA repair	11	MRE11A (rs604845)	DNA repair	11	0.0005	0.0013
0.0126	0.0129	<.0001	0.0014	INSR (rs2252673)	IIS	19	IDE (rs7078413)	IIS	10	0.0001	0.0002
0.0120	0.0127	<.0001	0.0014	EXO1 (rs1635518)	DNA repair	1	KL (rs9527026)	IIS	13	0.0003	0.0004

Further reducing the significance level, by selecting a *p *< .0001, we prioritized 22 top‐ranked synergies among SNPs, significantly enriched in longevity (Table 1). The best combination (FDR <0.0001) between SNPs in different genes was between *TXNRD1‐*rs17202060 and *TP53‐*rs2078486, respectively, belonging to the pro/antioxidant and DNA repair pathway. The second‐best interaction was found among *IGF1R‐*rs12437963 and *PTPN1‐*rs6067484, both belonging to the IIS pathway, and the third among genes *TP53‐*rs2078486 and *ERCC2‐*rs50871, both belonging to the DNA repair pathway.

Less significant interactions were found between *TP53* and two genes from the same DNA repair pathway: *TP53 (*rs2078486)*‐PON2* (rs2299267) and *TP53 (*rs2078486)*‐RECQL* (rs2284392) (FDR* *= 0.0032 and 0.0038, respectively); although not holding an FDR<0.005, they suggest a central role of TP53 in mediating the concerted activation of DNA repair and pro‐antioxidant pathways, as for example in stressful conditions.

### 2.2 MDR analysis

SNPsyn gave us a whole picture of the significantly interacting SNPs in the whole data set available for our study; however, that approach does not include covariates in the analysis, such as sex or age, simply dividing the data in cases and controls. Thus, to improve the case–control analysis of SNP‐SNP interactions, a MDR approach was applied. Table 2 reports the list of two‐ and three‐loci interactions prioritized by MDR analysis as the most significant for consistency value (minimum 2/10 replicates) and accuracy level (>0.5), between genes belonging to the same pathway and in the whole data set (last rows).

**Table 2:** Significant results of SNP‐SNP associations with longevity, resulting by MDR analysis. Two‐ and three‐loci interactions are shown, with respective pathways, prioritized for CV consistency and training balanced accuracy (> 0.5)

SNP combination	Genes included in the best combination	Pathways involved	High‐risk combination for longevity	OR (95% C.I.)	*p*‐value of interaction analysis (case–control study) by MDR	*p*‐value of survival analysis (Danish 1905 cohort)	
						Males	Females
rs572169(1)/rs512692(2)	*GHSR/GHSR*	IIS	SNP1‐GG/SNP2‐T carriers	0.61 (0.50–0.97)	.001	.961 (*N *= 285)	.070 (*N *= 715)
rs572169(1)/rs6067484(2)	*GHSR/PTPN1*	IIS	SNP1‐GG/SNP2‐AG	0.52 (0.42–0.66)	.001	.192 (*N *= 291)	.431 (*N *= 727)
rs572169(1)/rs512692(2)/rs4837525(3)	*GHSR/GHSR/PAPPA*	IIS	SNP1‐GG/SNP2‐TT/SNP3‐AG	0.86 (0.68–1.09)	.001	.716 (*N *= 282)	.262 (*N *= 709)
rs50871(1)/rs2078486(2)	*ERCC2/TP53*	DNA repair	SNP1‐C carriers/SNP2‐G carriers	0.80 (0.64–1.0)	.001	.746 (*N *= 308)	.946 (*N *= 757)
rs170548(1)/rs2436514(2)	*ATM/LONP1*	DNA repair	SNP1‐A carriers/SNP2‐AG	0.67 (0.56–0.82)	.002	.722 (*N *= 300)	.912 (*N *= 728)
rs2236270(1)/rs162557(2)/rs3756704(3)	*GSS/ CYP1B1/GLRX*	Pro/Antioxidant	SNP1‐AC/SNP2‐GG/SNP3‐AG	0.69 (0.52–0.91)	.001	.514 (*N *= 299)	.508 (*N *= 747)
rs572169(1)/rs533984(2)	*GHSR*‐*MRE11A*	IIS‐DNA repair	SNP1‐GG/SNP2‐G carriers	0.55 (0.45–0.67)	.001	.636 (*N *= 288)	.026 (*N *= 724)
rs572169(1)/rs533984(2)/rs225119(3)	*GHSR*/*MRE11A/PARK7*	IIS‐DNA repair‐pro/antioxidant	SNP1‐GG/SNP2‐G carriers/SNP3‐G carriers	0.53 (0.43–0.65)	.001	.340 (*N *= 285)	.096 (*N *= 717)

High‐risk genetic combinations for longevity resulting from MDR approach was tested for involvement in survival (in the oldest cohort), by analysing carriers of specific candidate combinations, respect to the other genotypes, for the sample stratified by sex. Odds ratio with 95% confidence interval (C.I.) was determined by logistic regression applied to candidate SNP combinations that were reported. *p*‐value of MDR was calculated by permutation analysis, while those reported for survival are referred to the log‐rank (Mantel–Cox) test.

#### 2.2.1 MDR: Analysis intrapathways

**Figure 2:**
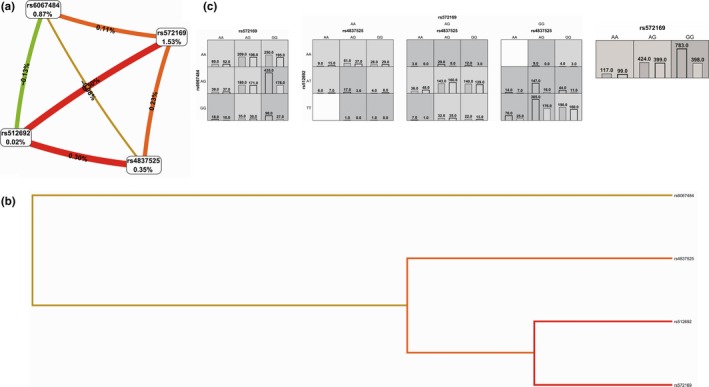
Interaction graph (a) and interaction dendrogram (b) for the IIS data set, resulting from MDR analysis. In a, for each SNP is reported in per cent the value of information gain (IG), while numbers in the connections indicate the entropy‐based IG for the SNP pairs. Red bar and orange bar indicate the high‐level synergies on the phenotype, while the brown indicate a medium‐level interaction, green and blue connections with negative IG values indicate redundancy or lack of synergistic interactions between the markers. Histograms in (c) reports the distributions of cases (left bars) and controls (right bars) for three‐locus and two‐locus genotype combinations of SNPs. The effect of rs572169(*GHSR* gene) is showed alone, because of its univariate effect. Dark‐shaded cells are considered “high risk,” while light‐shaded cells are considered “low risk” for longevity. White cells indicate no subjects with those genotype combinations that were observed in the data set

**Figure 3:**
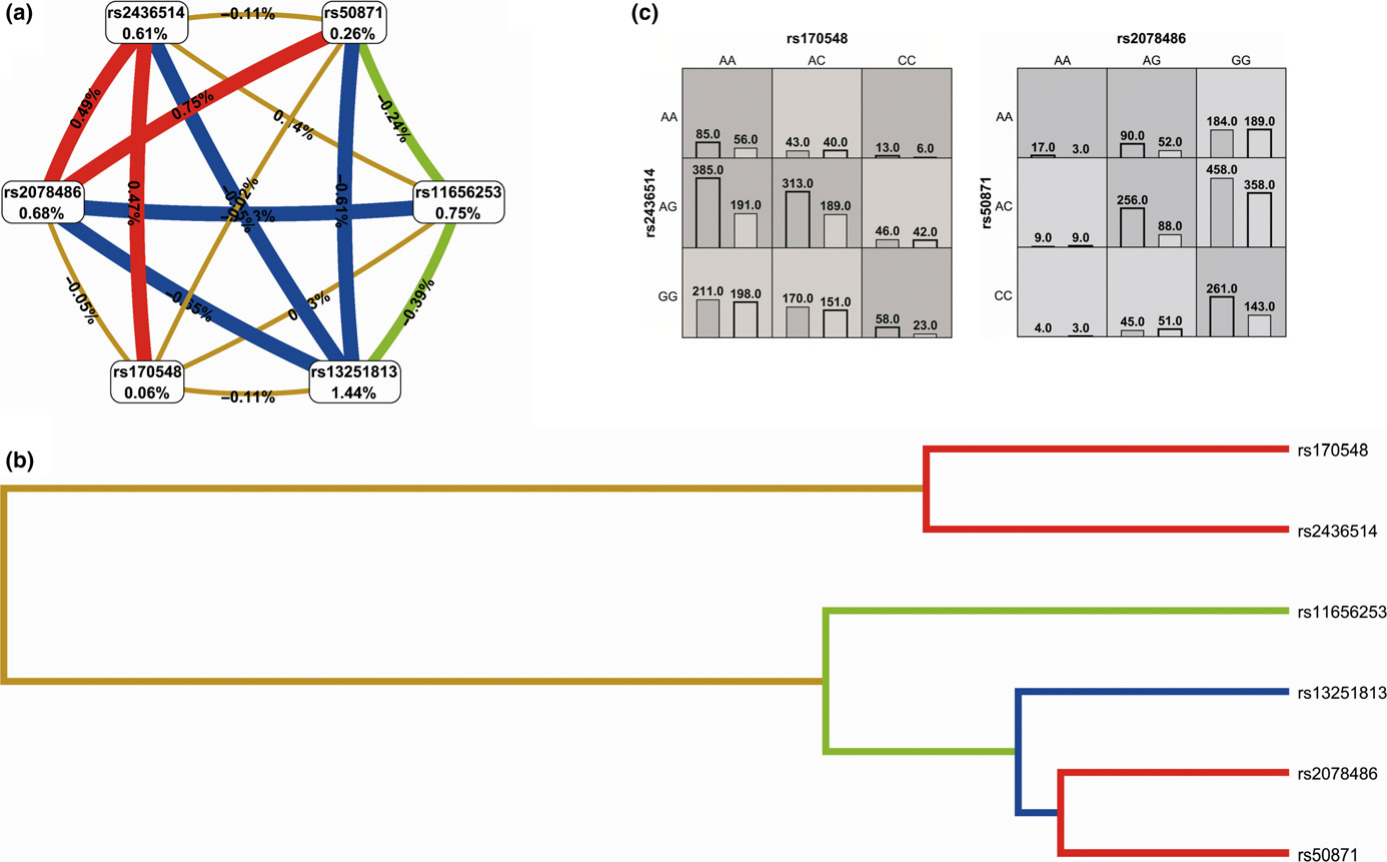
Interaction graph (a) and interaction dendrogram (b) for the DNA repair data set, resulting from MDR analysis. In a, for each SNP is reported in per cent the value of information gain (IG), while numbers in the connections indicate the entropy‐based IG for the SNP pairs. Red bar and orange bar indicate the high‐level synergies on the phenotype, while the brown indicate a medium‐level interaction, green and blue connections with negative IG values indicate redundancy or lack of synergistic interactions between the markers. Histograms in (c) report the distributions of cases (left bars) and controls (right bars) for three‐locus and two‐locus genotype combinations of SNPs. The effect of rs13251813 (*WRN* gene) is shown alone, because of its univariate effect. Dark‐shaded cells are considered “high risk,” while light‐shaded cells are considered “low risk.” White cells indicate no subjects with those genotype combinations that were observed in the data set

**Figure 4:**
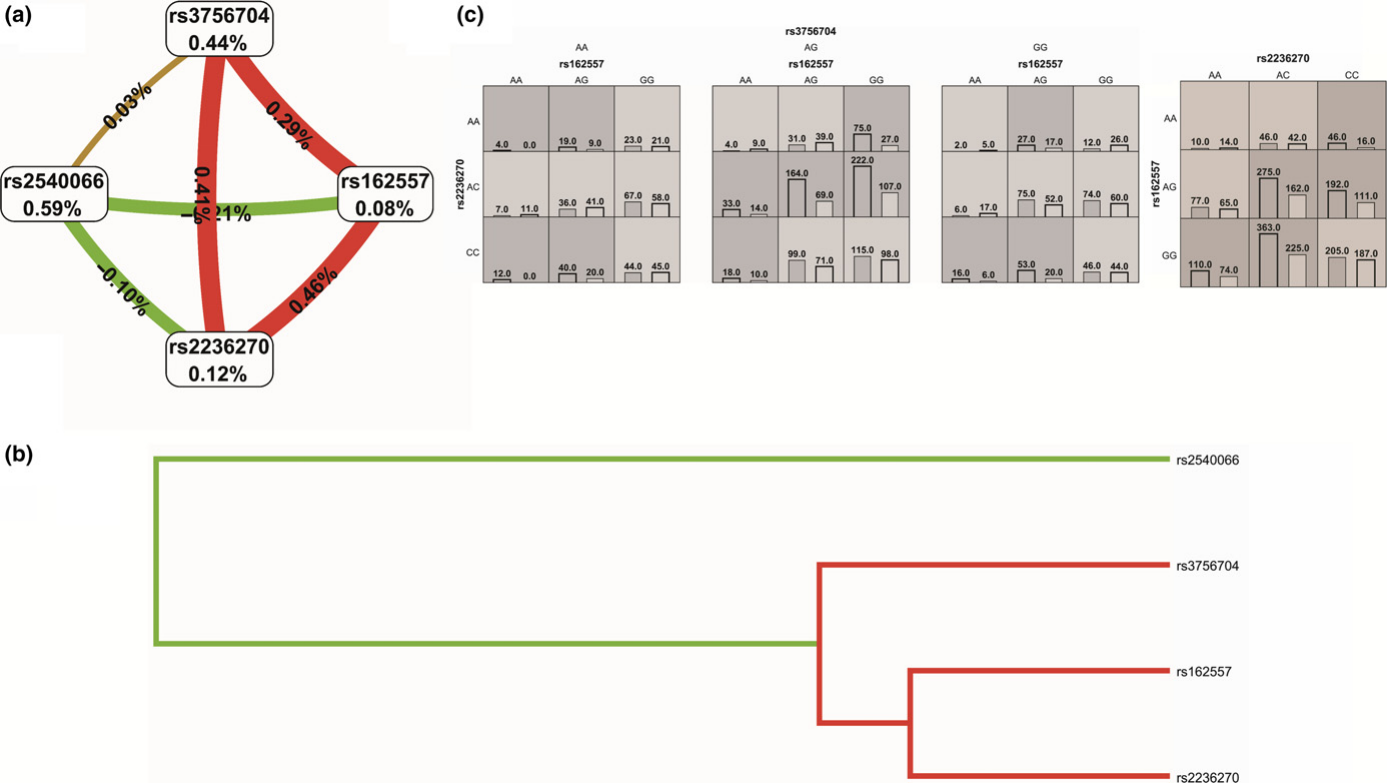
Interaction graph (a) and interaction dendrogram (b) for the pro/antioxidant data set, resulting from MDR analysis. In a, for each SNP is reported in per cent the value of information gain (IG), while numbers in the connections indicate the entropy‐based IG for the SNP pairs. Red bar and orange bar indicate the high‐level synergies on the phenotype, while the brown indicate a medium‐level interaction, green and blue connections with negative IG values indicate redundancy or lack of synergistic interactions between the markers. Histograms in (c) reports the distributions of cases (left bars) and controls (right bars) for three‐locus and two‐locus genotype combinations of SNPs. Dark‐shaded cells are considered “high‐risk” while light‐shaded cells are considered “low risk.” White cells indicate no subjects with those genotype combinations that were observed in the data set

First, the MDR confirmed the SNPsyn approach for what concerns the most significant SNP‐SNP interactions. The SNP‐SNP combination rs572169‐rs512692 inside the gene *GHSR* significantly interact with rs4837525 in the *PAPPA* gene inside the IIS pathway. rs572169‐*GHSR* also showed an interesting interaction with rs606748‐*PTPN1*. As indicated by the interaction graph (a), interaction dendrogram (b) and histograms (c) in Figure 2, these SNP‐SNP combinations jointly explain the most entropy, with the rs572169 SNP having the largest univariate effect (1.53% of explained entropy in the network), and the GG genotype driving the high‐risk combinations for longevity. The performance evaluation of each model (Table 2) shows that the two‐locus epistatic interaction performs better in terms of modelling (higher CV) compared to three‐locus interactions in these pathways. In the DNA repair sub‐data set (Figure 3), two high‐level epistatic interactions were found: one between rs50871 (*ERCC2* gene) and rs2078486 (*TP53*), and another between rs170548 (*ATM*) and rs2436514 (*LONP1*). Although the two indicated SNP‐SNP interactions reported low CV (Table 2), they were both significant by permutation analysis (*p *< .002). Interestingly, the *ERCC2‐TP53* interaction was highly significant also in SNPsyn analyses (FDR <.001 and *p *< .001), thus indicating a consistent association across methods of this SNP‐SNP combination with longevity. The largest univariate effect on longevity for the DNA repair pathway was recognized to rs13251813 (*WRN* gene), with 1.44% of explained entropy in the network (Figure 3). In the pro‐antioxidant data set, a three‐level SNP combination was found significantly associated with longevity among rs2236270 (*GSS* gene)‐ rs162557 (*CYP1B1* gene)‐rs3756704‐*GLRX* (*p *< .001) (Table 2 and Figure 4).

#### 2.2.2 MDR: Analysis interpathways

**Figure 5:**
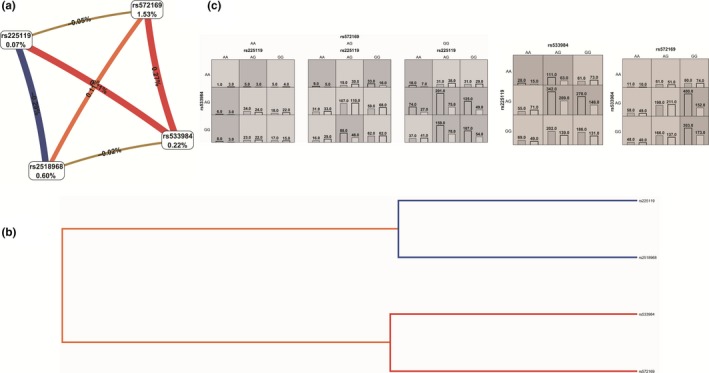
Interaction graph (a) and interaction dendrogram (b) for the DNA whole genetic data set, resulting from MDR analysis. In a, for each SNP is reported in per cent the value of Information Gain (IG), while numbers in the connections indicate the entropy‐based IG for the SNP pairs. Red bar and orange bar indicate the high‐level synergies on the phenotype, while the brown indicate a medium‐level interaction, green and blue connections with negative IG values indicate redundancy or lack of synergistic interactions between the markers. Histograms in (c) reports the distributions of cases (left bars) and controls (right bars) for three‐locus and two‐locus genotype combinations of SNPs. Dark‐shaded cells are considered “high‐risk” while light‐shaded cells are considered “low risk.” White cells indicate no subjects with those genotype combinations that were observed in the data set

Figure 5 shows the interaction graph (a) and interaction dendrogram (b) for the whole genotypic data set, as resulting from MDR analysis. In this case, we can see whether there are significant epistatic interactions between genes belonging to different metabolic biological pathways, among those investigated in this article. A significant epistasis was found between rs572169 (*GHSR* gene) belonging to the IIS pathway, which still has the largest univariate effect on the longevity phenotype (1.53% explained entropy), and rs533984 (*MRE11A* gene), from the DNA repair pathway. rs533984 positively interacts with rs225119 (*PARK7*), which belongs to the pro‐antioxidant pathway. Both the two‐locus interaction *GHSR‐MRE11A* and the three‐locus combination with *PARK7* were found to be significant (*p *= .001, Table 2); this may still depend on the higher univariate effect on longevity of the rs572169 SNP at the *GHSR* gene, with homozygotes GG favoured for longevity compared to the other genotypic combinations both in three‐ and two‐locus models.

### 2.3 Survival analysis

**Figure 6:**
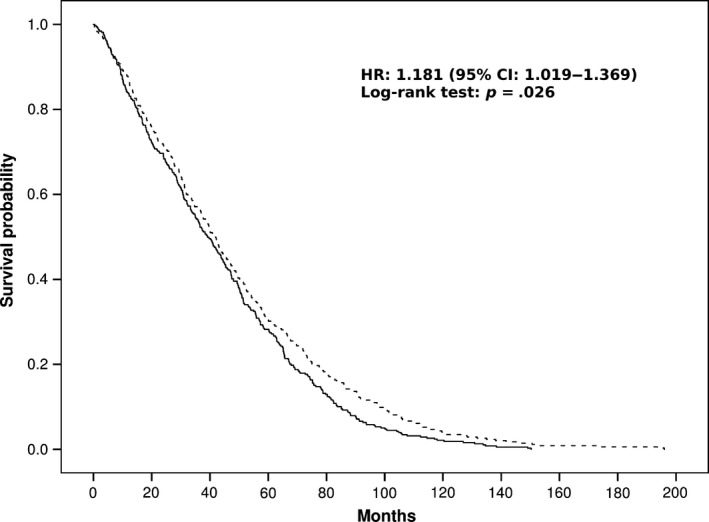
Survival function of female carriers of the protective combination rs572169(GG)‐rs533984(G/‐) (dashed line) vs. the cumulative survival function of all those carrying the other genotypic combinations at this loci (solid line). Time is expressed in months, where 0 is considered the time of recruitment, and each individual is followed up for his/her survival status till death. *p*‐value refers to log‐rank (Mantel–Cox) test. HR value and its confidence interval from the Cox regression analysis are reported inside the figure

Based on the information about postsurvey mortality available for the oldest cohort, the Danish 1905, we tested two‐ and three‐locus interactions significantly associated with longevity status in the previous analyses. With this analysis, we aimed to test whether those combinations, associated with an advantage in older individuals compared to younger subjects, were also favourable for survival at very old ages. Thus, we classified the subjects from the 1905 Danish cohort as carriers or noncarriers of a candidate risk combination for longevity and tested their survival as number of months survived from the recruitment. We performed survival analysis separately in male and female cohorts, considering the great importance of sex in survival differences inside a human population (reviewed in Alberts et al., 2014). As indicated in Table 2, among all relevant combinations tested, only that between rs572169 (*GHSR*) and rs533984 (*MREA11*) was found significantly associated with extreme survival in females (Figure 6) (*p *= .026, log‐rank Mantel–Cox test). In males, no effect on survival was observed for any of the candidate risk combinations of different SNPs.

## 3 DISCUSSION

Many reasons for the missing replicability in genetic association studies of survival to ages ≥90 years have been suggested, as it was recently reviewed by Sebastiani and coworkers, who indicated demographical reasons, that is the definition of the age windows for survival for birth cohort, as the main bias source when comparing different association studies on human longevity (Sebastiani, Nussbaum, Andersen, Black & Perls, 2016). Another possible explanation of the missing replicability and missing heritability may be related to the complexity of longevity (Dato et al., 2017), which harbours many heterogeneity sources, including the effect of rare variants, not captured by standard genomewide genotyping, and interactions between different loci, an often‐cited reason for the lack of success in genetic studies of complex diseases (Moore, 2003). With the aim of exploring this poorly investigated genetic effect, we re‐analysed a genetic data set previously described and used for single‐SNP and gene set analyses (Debrabant et al., 2014; Soerensen et al., 2012), for SNP‐SNP interactions. The findings obtained indeed indicate that an epistatic analysis approach is very much applicable for candidate gene/pathway data and hence might contribute to the knowledge concerning the genetics of human longevity.

First, we carried out SNP‐SNP association analysis by a classical case–control approach; that is, we compared the oldest‐old and youngest individuals for the frequency of relevant SNP pairs. We chose the SNPsyn software among the various existing tools reviewed in Cordell (2009), because it directly calculates the amount of synergy, also reflecting a balanced effect of two variants with equal importance. The most interesting findings of this analysis were the epistatic interactions intergenes (*TP53*‐DNA repair pathway/*TXNRD1‐*pro‐antioxidant pathway and *TP53*‐DNA repair pathway/*ERCC2*‐DNA repair pathway). Furthermore, while the interaction analysis confirmed the association of rs572169 of *GHSR* reported in a previous study using single‐SNP‐analysis (Soerensen et al., 2012), single variants in *TP53*,* TXNRD1* or *ERCC2* here identified were not previously found. In particular, a central role of *TP53* might not seem surprising as TP53 is a very well‐known tumour suppressor in DNA damage response which balances tumour surveillance and maintenance of stem cell pools, finally resulting in beneficial effects both for cancer protection and longevity (Reinhardt & Schumacher, 2012). In addition, the strong interaction with *TXNRD1*, recently found associated with the quality of aging (Dato, De Rango, Crocco, Passarino & Rose, 2015), suggests a central role of *TP53* in mediating the concerted activation of DNA repair and the pro‐antioxidant pathway. *TP53* has been suggested to exert longevity assurance functions by impacting on the conserved IGF‐1 system (Feng & Levine, 2010), yet we did not find interactions at genetic level among *TP53* and genes belonging to the IIS pathway in this study population.

According to the MDR analysis, the *GHSR* gene has an important univariate effect on longevity in the Danish study population; the analyses suggest synergistic interactions, favourable for longevity, with both the *PAPPA* and *PTPN1* genes from the same IIS pathway in longevity, but also with the *MRE11A* gene from the DNA repair and *PARK7* gene from the pro‐antioxidant pathway. Survival analysis in the oldest‐old cohort demonstrated that the interaction between *GHSR* and *MRE11A* (rs572169/rs533984) has a positive effect on female survival (*p *= .026), with carriers of the combination rs572169‐GG /rs533984‐G showing a less mortality. Interestingly, *MRE11A*, according to SNPsyn analysis, is part of a network with *IGFIR*,* PTPN*1 and *MSH3*. This multiple level interaction highlights the interaction between the IIS pathway (*IGF1R* and *PTPN1*) and DNA repair (*MRE11A* and *MSH3*). The link between the IGF1R‐PTPN1 proteins is known: literature data indicate a crucial role of PTPN1 as a tumour suppressor, able to negatively regulate multiple pathways of cell growth directly through *IGF1R* and *IR*, or indirectly through leptin receptor GHR (Neel & Tonks, 2016). Experimental data report that the protein interaction IGF1R‐PTPN1 is regulated by insulin levels (Bandyopadhyay et al., 1997). Furthermore, in experimental organisms, *PTPN1* is involved in inflammatory mechanisms and insulin resistance associated with diabetes and obesity during aging (González‐Rodríguez et al., 2012). Thus, although still not studied in relation to successful aging, the observed interaction among genetic variants of *IGF1R* and *PTPN1* in Danish long‐lived could reflect a conserved mechanism of cellular signalling, involved in the development of metabolic diseases and tumorigenesis.

In conclusion, the results here presented illustrate the validity of interaction approach and network analyses for investigating the genetic contribution in human longevity. The analyses not only confirm a gene previously identified by single‐SNP‐analysis in the same data set (*GHSR*), but found novel interaction partners of such gene (*PAPPA*,* PTPN1*,* MRE11A* and *PARK7*). Furthermore, this methodology identifies novel genes as central nodes of additional networks (e.g., *TP53*) involved in human longevity. Obviously, these interesting findings will need further validation, possibly by the confirmation of top SNP‐SNP interactions found in this work independently replicated in additional study populations. Moreover, functional analyses (expression studies and protein interaction testing) should be implemented, to determine the biological relevance of the statistical interactions.

## 4 EXPERIMENTAL PROCEDURES

### 4.1 Study population and genetic data set

The characteristics of the study population were elsewhere reported (Soerensen et al., 2012) and summarized in Table S2 (Supplementary materials). Briefly, the sample studied here included 1,089 cases (mean age: 93.1 years, age range 92.2–93.8 years, 29% males and 71% females) drawn from eligible members of the Danish 1905 Birth Cohort Study (Nybo et al., 2001) and 736 controls (mean age 50.6, age range 46.0–55.0, gender distribution 50% males and 50% females) from the Study of Middle Aged Danish Twins (MADT) (Skytthe et al., 2013). Information on survival status per 1st of January 2015 for the oldest‐old was retrieved from the Danish Central Population Register (Pedersen, Gotzsche, Moller & Mortensen, 2006). Permission to collect blood samples and to use survey information was granted by The Danish Regional Committees on Biomedical Research Ethics.

The genotype data set and genotyping methodology by the Illumina GoldenGate platform has been described in previous papers (Soerensen et al., 2010, 2012). To exclude the rare variants, a minimum allele frequency (MAF) level of 5% was chosen for performing the analyses, leaving 1,058 SNPs from the original data set to test SNP‐SNP associations with longevity. A table reporting all SNPs investigated can be found in the Supplementary material (Table S3), classified by gene and pathway. In detail, 317 SNPs were belonging to the IIS pathway, 260 to pro‐antioxidant response, 481 to DNA damage signalling and repair. Furthermore, functional prediction of most significant SNPs in SNP pairs found to be associated with longevity, based on Genome Browser information (Haploreg, http://archive.broadinstitute.org/mammals/haploreg/haploreg.php) was reported in Table S4.

### 4.2 Statistical methodology

Genotype counts for testing HWE were performed in the control group sample (Li & Li, 2008; Schaid, Batzler, Jenkins & Hildebrandt, 2006) by Plink (Purcell et al., 2007), choosing a cutoff of 0.001 for excluding SNPs not in HWE in the control sample. Minor allele frequency (MAF) was computed for each SNP, and differences in genotype frequency between cases controls were calculated by RobustSNP (So & Sham, 2011) in the R environment (https://wwwr-project.org/). As reported in Soerensen et al., 2012; tagging SNPs were prioritized by establishing a cutoff of *r* ^2^ **= .8, LOD* *= 3, minor allele frequency (MAF)>5% and a minimum distance between SNPs* *= 60 bp criteria. The selection of common variants (MAF ≥ 5) and a LD *r* ^2^ < .8 is widely used in genomewide association studies (Fadista, Manning, Florez & Groop, 2016). Furthermore, we tested LD levels by genomic region in the control sample (data not shown).

### 4.3 SNPsyn analysis of SNP‐SNP interactions

SNP‐SNP interactions significantly differing in frequency between cases and controls were computed by SNPsyn, according to the method described in Curk et al., 2011; which allows the discovery of synergistic pairs of SNPs from large data sets in association studies with complex traits. Synergy (Syn) among SNPs was estimated by an information‐theoretic approach, without specifying interaction models, but assuming an additive model, where the expected amount of information on the phenotype for a combination of SNPs is equal to the sum of information of the individual SNPs. For each SNP‐SNP combination, information gain (IG) and entropy were calculated, which corresponds to the level of association between markers and phenotype. Pairs of SNPs with positive synergy (Syn>0) were called synergistic; negative synergy (Syn<0) indicates that the two SNPs carry redundant information, an effect typically observed among highly correlated SNPs in LD. Information about pathway membership by Gene Ontology was used for performing enrichment analysis on subpathways, for genes associated with the phenotype. Obtained significance scores were corrected for multiple testing using the false discovery rate (FDR) method described by Benjamini and Yekutieli (2001). In this paper, significant associations were considered those reporting a FDR< 0.005 (*p*‐value <.0001). Because FDR is a measure of the per cent of false‐positive results, and it is inversely proportional to the number of SNPs tested, we chose a stringent level to minimize the false‐positive combinations in our analysis.

Interaction analyses were computed online, at the website http://snpsyn.biolab.si/.

### 4.4 Gene set enrichment analysis

Gene set enrichment analysis was performed by SNPsyn on the whole data set to find biological processes in which represented genes were involved in human longevity (based on the known information about their gene products (data driven by Gene Ontology)). Which gene sets to test were determined by SNPsyn based on significant association with the phenotype (as previously explained). Subsequently, the gene sets were used as a cluster (query) set in this gene enrichment analysis, with the goal to determine whether members of a gene set tend to occur towards the top (or bottom) of the list. In such case, the gene set was correlated with the phenotypic class distinction. Fold enrichment score reflects the degree to which a set is overrepresented at the extremes (top or bottom) of the entire ranked list.

### 4.5 MDR analysis of epistatic interactions

For testing the epistatic interaction between pairs of SNPs, multifactor dimensionality reduction (MDR) was applied (Moore, 2004; Ritchie et al., 2001). This methodology can reveal high‐order interactions among genes collaborating with respect to a given phenotype, with the aim of determining multilocus genotype combinations associated with high or low risk of disease. The entropy‐based clustering algorithm used by MDR sets a contingency table for k SNPs and calculates case–control ratios for each of the possible multilocus genotypes. Hence, a genotype combination is considered high‐level if more present in cases respect to controls. The MDR interaction model describes percentage of entropy (information gain or IG) by each factor (values in the nodes indicate independent main effect) or 2‐way interaction. Graphical visualization through connections among the markers help to interpret additive and nonadditive interactions effects on phenotype: positive values of entropy indicate synergistic or nonadditive interaction while negative entropy values indicate redundancy between the markers or lack of any synergistic interaction between the markers. As for colours, connections in red and orange indicate nonlinear interaction, connections in green and brown indicate independence or additivity and redundancy (blue lines). In a data set of more than two interacting factors, the best model is considered the one found more consistent in different replicates (expressed as CV, consistency values and accuracy, i.e. training balanced accuracy level). For calculating significance, permutation testing is applied, dividing the data set into 10 portions, and using nine portions as a training data set, and the remaining as a testing data set. One thousand permutations were performed, to determine a cutoff threshold for an α = 0.05 significance level. Missing genotypes were imputed with the MDR data tool software (version 0.4.3), which allows the global replacement of unknown values and the imputation of the data from a model constructed by the software from the existing data set.

MDR analyses were implemented in the open‐source MDR software package version 3.0.2 (available on http://www.multifactordimensionalityreduction.org/).

### 4.6 Survival analysis of relevant SNP‐SNP interactions

Kaplan–Meier survival curves with the Mantel–Cox log‐rank test were used to compare survival of oldest‐old subjects with different SNP‐SNP combinations (Therneau & Grambsch, 2000). Survival analyses were performed using the survival package in R, as described in Therneau (2005). A significance level of 0.05 was chosen in all tests.

## AUTHOR CONTRIBUTIONS

Serena Dato drafted the manuscript and generated the conception of the study, and participated in the data acquisition and interpretation of data. Mette Soerensen generated the study design and participated in the data acquisition, data management and interpretation of data. Francesco De Rango carried out the statistical analyses and participated in the interpretation of data. Giuseppina Rose participated in the interpretation of data and revision of the manuscript. Lene Christiansen participated in generating the study design, acquisition of data and interpretation of data. Giuseppe Passarino participated in the interpretation of data and revision of the manuscript. Kaare Christensen participated in the acquisition of data and interpretation of data. All authors have revised the manuscript and given their final approval.
