# Supplementary material for: The genetic component of human longevity: New insights from the analysis of pathway‐based SNP‐SNP interactions
Source: Aging Cell. 2018 Mar 25;17(3):e12755. doi: 10.1111/acel.12755 (PMC5946073; doi:10.1111/acel.12755)
Supplement: Supplementary file 1 [file ACEL-17-e12755-s001.docx]

| Syn | I | P-value | FDR | Snp 1 | Chr 1 | **Pathway** | Snp 2 | Chr 2 | | **Pathway** | I 1 | | I 2 |
| --- | --- | --- | --- | --- | --- | --- | --- | --- | --- | --- | --- | --- | --- |
| 0.0205 | 0.0325 | <0.0001 | <0.0001 | *GHSR (rs512692)* | 3p95 | INS/IGF1 | *GHSR (rs572169)* | 3p951 | | INS/IGF1 | 0.0006 | | 0.0115 |
| 0.0199 | 0.0246 | <0.0001 | <0.0001 | *ERCC1 (rs3212961)* | 19q47 | DNA repair | *ERCC1 (rs762562)* | 19q47 | | DNA repair | 0.0034 | | 0.0013 |
| 0.0181 | 0.0297 | <0.0001 | <0.0001 | *GHRHR (rs2267723)* | 7p91 | INS/IGF1 | *GHRHR (rs4988505)* | 7p914 | | INS/IGF1 | 0.0112 | | 0.0004 |
| 0.0159 | 0.0214 | <0.0001 | <0.0001 | *PTPN1 (rs2038526)* | 20p31 | INS/IGF1 | *PTPN1 (rs6067484)* | 20p31 | | INS/IGF1 | 0.0001 | | 0.0054 |
| 0.0143 | 0.0178 | <0.0001 | <0.0001 | *ERCC1 (rs3212961)* | 19q47 | DNA repair | *ERCC1 (rs3212964)* | 19q47 | | DNA repair | 0.0034 | | 0.0001 |
| 0.0140 | 0.0182 | <0.0001 | <0.0001 | *NBN (rs12680687)* | 8p91 | DNA repair | *NBN (rs2735385)* | 8p91 | | DNA repair | 0.0041 | | 0.0001 |
| 0.0127 | 0.0147 | <0.0001 | 0.0012 | *PRDX3 (rs1553850)* | 10q87 | Pro/Antioxidant | *PAPPA (rs449807)* | 9q916 | | INS/IGF1 | 0.0010 | | 0.0011 |
| 0.0126 | 0.0129 | <0.0001 | 0.0014 | *INSR (rs2252673)* | 19p73 | INS/IGF1 | *IDE (rs7078413)* | 10q11 | | INS/IGF1 | 0.0001 | | 0.0002 |
| 0.0126 | 0.0182 | <0.0001 | <0.0001 | *PTPN1 (rs2426164)* | 20p31 | INS/IGF1 | *PTPN1 (rs6067484)* | 20p31 | | INS/IGF1 | 0.0002 | | 0.0054 |
| 0.0125 | 0.0165 | <0.0001 | <0.0001 | *XRCC1 (rs1799782)* | 19q74 | DNA repair | *XRCC1 (rs3213403)* | 19q74 | | DNA repair | 0.0005 | | 0.0035 |
| 0.0122 | 0.0139 | <0.0001 | 0.0012 | *MRE11A (rs10831227)* | 11q30 | DNA repair | *MRE11A (rs604845)* | 11q30 | | DNA repair | 0.0005 | | 0.0013 |
| 0.0120 | 0.0127 | <0.0001 | 0.0014 | *EXO1 (rs1635518)* | 1q10 | DNA repair | *KL (rs9527026)* | 13p31 | | INS/IGF1 | 0.0003 | | 0.0004 |
| 0.0120 | 0.0189 | <0.0001 | <0.0001 | *TXNRD1 (rs17202060)* | 12p24 | Pro/Antioxidant | *TP53 (rs2078486)* | 17p73 | | DNA repair | 0.0007 | | 0.0062 |
| 0.0113 | 0.0136 | <0.0001 | 0.0016 | *PON1 (rs2074351)* | 7q94 | Pro/Antioxidant | *PON1 (rs662)* | 7q94 | | Pro/Antioxidant | 0.0017 | | 0.0006 |
| 0.0112 | 0.0196 | <0.0001 | <0.0001 | *IGF1R (rs12437963)* | 15p31 | INS/IGF1 | *PTPN1 (rs6067484)* | 20p31 | | INS/IGF1 | 0.0030 | | 0.0054 |
| 0.0112 | 0.0186 | <0.0001 | <0.0001 | *TP53 (rs2078486)* | 17p73 | DNA repair | *ERCC2 (rs50871)* | 19q47 | | DNA repair | 0.0062 | | 0.0012 |
| 0.0110 | 0.0124 | <0.0001 | 0.0037 | *PON2 (rs12026)* | 7q94 | Pro/Antioxidant | *PON2 (rs17879277)* | 7q94 | Pro/Antioxidant | | 0.0007 | 0.0007 | |
| 0.0108 | 0.0131 | <0.0001 | 0.0037 | *RAD50 (rs2706370)* | 5p16 | DNA repair | *NEIL2 (rs804269)* | 8p91 | | DNA repair | 0.0020 | | 0.0003 |
| 0.0107 | 0.0130 | <0.0001 | 0.0037 | *GCLC (rs7742367)* | 6p33 | *INS/IGF1* | *XRCC4 (rs1011980)* | 5p16 | | DNA repair | 0.0010 | | 0.0013 |
| 0.0107 | 0.0120 | <0.0001 | 0.0046 | *PARK7 (rs225092)* | 1p37 | Pro/Antioxidant | *PARK7 (rs226242)* | 1p37 | | Pro/Antioxidant | 0.0005 | | 0.0007 |
| 0.0106 | 0.0114 | <0.0001 | 0.0046 | *AOX1 (rs2002957)* | 2q91 | Pro/Antioxidant | *KL (rs687045)* | 13p31 | | *INS/IGF1* | 3,81E-01 | | 0.0007 |
| 0.0106 | 0.0130 | <0.0001 | 0.0037 | *TXNRD1 (rs10778318)* | 12p24 | Pro/Antioxidant | *ERCC5 (rs4150355)* | 13q38 | | DNA repair | 0.0019 | | 0.0005 |
| 0.0105 | 0.0110 | <0.0001 | 0.0046 | *EXO1 (rs1635518)* | 1q10 | DNA repair | *KL (rs9536314)* | 13p31 | | INS/IGF1 | 0.0003 | | 0.0002 |
| 0.0105 | 0.0132 | <0.0001 | 0.0046 | *FOXO3 (rs2802292)* | 6p33 | INS/IGF1 | *INSR (rs2860184)* | 19p73 | | INS/IGF1 | 0.0009 | | 0.0019 |
| 0.0102 | 0.0165 | <0.0001 | 0.0006 | *PTPN1 (rs6063534)* | 20p31 | INS/IGF1 | *PTPN1 (rs6067484)* | 20p31 | | INS/IGF1 | 0.0008 | | 0.0054 |
| 0.0101 | 0.0123 | <0.0001 | 0.0046 | *FANCA (rs7190823)* | 16p18 | DNA repair | *IGFBP2 (rs3770473)* | 2q73 | | INS/IGF1 | 0.0019 | | 0.0002 |
| 0.0100 | 0.0126 | <0.0001 | 0.0046 | *POLG (rs3176208)* | 15q49 | DNA repair | *IGF1R (rs7168671)* | 15p31 | | INS/IGF1 | 0.0023 | | 0.0002 |
| 0.0100 | 0.0126 | <0.0001 | 0.0046 | *PON1 (rs2299261)* | 7q94 | Pro/Antioxidant | *TXNRD1 (rs4964778)* | 12p24 | | Pro/Antioxidant | 0.0007 | | 0.0019 |
| 0.0099 | 0.0141 | <0.0001 | 0.0020 | *NBN (rs12680687)* | 8p91 | DNA repair | *RAD50 (rs2237060)* | 5p16 | | DNA repair | 0.0041 | | 3,79E-01 |
| 0.0098 | 0.0125 | <0.0001 | 0.0046 | *TXNRD1 (rs4964728)* | 12p24 | Pro/Antioxidant | *IDE (rs2421943)* | 10q11 | | INS/IGF1 | 6,76E-01 | | 0.0027 |
| 0.0098 | 0.0157 | <0.0001 | 0.0006 | *RPA1 (rs11656253)* | 17p73 | INS/IGF1 | *RPA1 (rs17292175)* | 17p73 | | INS/IGF1 | 0.0058 | | 9,61E-01 |
| 0.0097 | 0.0155 | <0.0001 | 0.0006 | *MRE11A (rs512150)* | 11q30 | DNA repair | *MRE11A (rs592068)* | 11q30 | | DNA repair | 0.0041 | | 0.0017 |
| 0.0097 | 0.0123 | <0.0001 | 0.0046 | *XPC (rs2733534)* | 3p95 | DNA repair | *IGF1R (rs4965438)* | 15p31 | | INS/IGF1 | 0.0008 | | 0.0018 |
| 0.0096 | 0.0138 | <0.0001 | 0.0020 | *RAD23B (rs11573709)* | 9q31 | DNA repair | *EXO1 (rs12118937)* | 1q10 | | DNA repair | 0.0041 | | 4,48E-01 |
| 0.0096 | 0.0127 | <0.0001 | 0.0046 | *LIG4 (rs1805388)* | 13q91 | DNA repair | *NEIL2 (rs8191534)* | 8p91 | | DNA repair | 0.0013 | | 0.0019 |
| 0.0095 | 0.0145 | <0.0001 | 0.0020 | *NBN (rs3026271)* | 8p91 | DNA repair | *INSR (rs11672739)* | 19p73 | | INS/IGF1 | 0.0012 | | 0.0038 |
| 0.0094 | 0.0123 | <0.0001 | 0.0046 | *ERCC1 (rs3212948)* | 19q47 | DNA repair | *IDE (rs6583820)* | 10q11 | | INS/IGF1 | 0.0025 | | 0.0004 |
| 0.0094 | 0.0121 | <0.0001 | 0.0046 | *H2AFX (rs2509049)* | 11q88 | DNA repair | *AKT1 (rs2494731)* | 14q32 | | INS/IGF1 | 0.0027 | | 1,40E-01 |
| 0.0093 | 0.0127 | <0.0001 | 0.0046 | *H2AFX (rs2509049)* | 11q88 | DNA repair | *AKT1 (rs2498796)* | 14q32 | | INS/IGF1 | 0.0027 | | 0.0007 |
| 0.0092 | 0.0142 | <0.0001 | 0.0020 | *GLRX (rs871775)* | 5p16 | INS/IGF1 | *IGF1 (rs1520220)* | 12p24 | | INS/IGF1 | 0.0037 | | 0.0013 |
| 0.0091 | 0.0156 | <0.0001 | 0.0006 | *AOX1 (rs2465661)* | 2q91 | Pro/Antioxidant | *RAD23B (rs11573709)* | 9q31 | | DNA repair | 0.0024 | | 0.0041 |
| 0.0091 | 0.0125 | <0.0001 | 0.0046 | *RECQL (rs10841833)* | 12p24 | DNA repair | *POLRMT (rs2283575)* | 19p73 | | DNA repair | 1,21E-01 | | 0.0034 |
| 0.0090 | 0.0120 | <0.0001 | 0.0046 | *RPA1 (rs2287320)* | 17p73 | INS/IGF1 | *IRS2 (rs2099435)* | 13p31 | | INS/IGF1 | 0.0005 | | 0.0025 |
| 0.0090 | 0.0149 | <0.0001 | 0.0020 | *BLM (rs3784782)* | 15q82 | DNA repair | *FOXO1 (rs2701858)* | 13p31 | | *INS/IGF1* | 0.0002 | | 0.0057 |
| 0.0088 | 0.0140 | <0.0001 | 0.0032 | *XDH (rs2043013)* | 2p90 | Pro/Antioxidant | *PARP1 (rs1136410)* | 1q10 | | DNA repair | 0.0003 | | 0.0048 |
| 0.0088 | 0.0149 | <0.0001 | 0.0032 | *TXNRD1 (rs10778318)* | 12p24 | Pro/Antioxidant | *XRCC1 (rs3213266)* | 19q74 | | DNA repair | 0.0019 | | 0.0042 |
| 0.0087 | 0.0161 | <0.0001 | 0.0009 | *WRN (rs11574218)* | 8p53 | DNA repair | *ERCC1 (rs3212961)* | 19q47 | | DNA repair | 0.0040 | | 0.0034 |
| 0.0087 | 0.0150 | <0.0001 | 0.0032 | *NOX1 (rs4828068)* | 23q43 | Pro/Antioxidant | *MSH3 (rs26279)* | 5p16 | | DNA repair | 0.0062 | | 8,74E-01 |
| 0.0086 | 0.0136 | <0.0001 | 0.0032 | *GLRX (rs3756704)* | 5p16 | Pro/Antioxidant | *IGF1 (rs10860865)* | 12p24 | | *INS/IGF1* | 0.0034 | | 0.0017 |
| 0.0086 | 0.0147 | <0.0001 | 0.0032 | *PON2 (rs2375005)* | 7q94 | Pro/Antioxidant | *BLM (rs2518968)* | 15q82 | | DNA repair | 0.0001 | | 0.0060 |
| 0.0086 | 0.0148 | <0.0001 | 0.0032 | [*CYP1B1*](https://www.ncbi.nlm.nih.gov/entrez/query.fcgi?db=gene&cmd=Retrieve&dopt=Graphics&list_uids=1545) *(rs162556)* | 2p57 | Pro/Antioxidant | *RAD23B (rs1805329)* | 9q31 | | DNA repair | 0.0003 | | 0.0059 |
| 0.0085 | 0.0139 | <0.0001 | 0.0032 | *GCLC (rs572496)* | 6p33 | *INS/IGF1* | *EXO1 (rs4149867)* | 1q10 | | DNA repair | 0.0038 | | 0.0015 |
| 0.0084 | 0.0158 | <0.0001 | 0.0009 | *AOX1 (rs2256977)* | 2q91 | Pro/Antioxidant | *KL (rs2283368)* | 13p31 | | *INS/IGF1* | 0.0039 | | 0.0035 |
| 0.0083 | 0.0146 | <0.0001 | 0.0032 | *DCLRE1C (rs12572872)* | 10p44 | DNA repair | *POLRMT (rs2283575)* | 19p73 | | DNA repair | 0.0029 | | 0.0034 |
| 0.0081 | 0.0154 | <0.0001 | 0.0009 | *PARP1 (rs1136410)* | 1q10 | DNA repair | *FOXO3 (rs479744)* | 6p33 | | *INS/IGF1* | 0.0048 | | 0.0024 |
| 0.0081 | 0.0146 | <0.0001 | 0.0032 | *PON2 (rs2299267)* | 7q94 | Pro/Antioxidant | *TP53 (rs2078486)* | 17p73 | | DNA repair | 0.0003 | | 0.0062 |
| 0.0079 | 0.0145 | <0.0001 | 0.0032 | *WRN (rs11574218)* | 8p53 | DNA repair | *PTEN (rs1903858)* | 10q11 | | *INS/IGF1* | 0.0040 | | 0.0026 |
| 0.0079 | 0.0150 | <0.0001 | 0.0032 | *MRE11A (rs512150)* | 11q30 | DNA repair | *IGF1R (rs12437963)* | 15p31 | | *INS/IGF1* | 0.0041 | | 0.0030 |
| 0.0078 | 0.0141 | <0.0001 | 0.0032 | *NOX1 (rs4828068)* | 23q43 | Pro/Antioxidant | *MSH3 (rs6151616)* | 5p16 | | DNA repair | 0.0062 | | 2,66E-01 |
| 0.0077 | 0.0135 | <0.0001 | 0.0032 | *PON2 (rs7803148)* | 7q94 | Pro/Antioxidant | *RPA1 (rs11656253)* | 17p73 | | *INS/IGF1* | 3,06E-02 | | 0.0058 |
| 0.0076 | 0.0146 | <0.0001 | 0.0032 | *POLG (rs3176208)* | 15q49 | DNA repair | *INSR (rs8110428)* | 19p73 | | *INS/IGF1* | 0.0023 | | 0.0047 |
| 0.0076 | 0.0144 | <0.0001 | 0.0032 | *TXNRD1 (rs10778318)* | 12p24 | Pro/Antioxidant | *XRCC1 (rs2023614)* | 19q74 | | DNA repair | 0.0019 | | 0.0049 |
| 0.0076 | 0.0137 | <0.0001 | 0.0032 | *GSR (rs1002149)* | 8p53 | Pro/Antioxidant | *IRS1 (rs6725330)* | 2q20 | | *INS/IGF1* | 0.0060 | | 0.0001 |
| 0.0075 | 0.0137 | <0.0001 | 0.0032 | *RPA1 (rs11656253)* | 17p73 | DNA repair | *RPA1 (rs4790830)* | 17p73 | | DNA repair | 0.0058 | | 0.0003 |
| 0.0075 | 0.0138 | <0.0001 | 0.0032 | *MSH3 (rs397628)* | 5p16 | DNA repair | *PTPN1 (rs6067484)* | 20p31 | | *INS/IGF1* | 0.0008 | | 0.0054 |
| 0.0075 | 0.0140 | <0.0001 | 0.0038 | *TP53 (rs2078486)* | 17p73 | DNA repair | *RECQL (rs2284392)* | 12p24 | | DNA repair | 0.0062 | | 0.0003 |
| 0.0073 | 0.0143 | <0.0001 | 0.0038 | *RPA1 (rs11656253)* | 17p73 | DNA repair | *MSH6 (rs3136329)* | 2p38 | | DNA repair | 0.0058 | | 0.0012 |
| 0.0073 | 0.0139 | <0.0001 | 0.0038 | *BLM (rs2518968)* | 15q82 | DNA repair | *IGF1R (rs3743258)* | 15p31 | | *INS/IGF1* | 0.0060 | | 0.0006 |
| 0.0072 | 0.0139 | <0.0001 | 0.0038 | *AOX1 (rs2256977)* | 2q91 | Pro/Antioxidant | *TXNRD1 (rs4964735)* | 12p24 | | Pro/Antioxidant | 0.0039 | | 0.0028 |
| 0.0072 | 0.0136 | <0.0001 | 0.0038 | *ERCC2 (rs1799788)* | 19q47 | DNA repair | *PTEN (rs17431184)* | 10q11 | | *INS/IGF1* | 0.0044 | | 0.0021 |
| 0.0071 | 0.0140 | <0.0001 | 0.0038 | *RAD23B (rs1805329)* | 9q31 | DNA repair | *IGF2R (rs3798187)* | 6p33 | | *INS/IGF1* | 0.0059 | | 0.0010 |
| 0.0070 | 0.0140 | <0.0001 | 0.0038 | *RAD23B (rs1805329)* | 9q31 | DNA repair | *IGF2R (rs9347380)* | 6p33 | | *INS/IGF1* | 0.0059 | | 0.0010 |
| 0.0068 | 0.0136 | <0.0001 | 0.0038 | *BLM (rs389480)* | 15q82 | DNA repair | *IDE (rs2421943)* | 10q11 | | *INS/IGF1* | 0.0040 | | 0.0027 |

Table 1S: List of top-ranked 73 SNP-SNP interactions, sorted by synergy level, resulting from SNPsyn analysis on the whole genotypic dataset [1824 samples (1088 long lived, 736 younger controls), 1058 SNPs] and significant according to FDR <0.005. Interactions intra-pathway: 11 INS/IGF1; 17 DNA-repair; 5 Pro-Antioxidant. Interactions among different pathways: 22 INS/IGF1-DNA repair; 11 Pro-Antioxidant-DNA repair; 7 Pro-Antioxidant-INS/IGF1. Syn: synergy estimate between SNP pairs, FDR: False Discovery Rate; I: information, I1 and I2: contribution of SNP1 and SNP2 to the synergic interaction, respectively.
