# Supplementary material for: The genetic component of human longevity: New insights from the analysis of pathway‐based SNP‐SNP interactions
Source: Aging Cell. 2018 Mar 25;17(3):e12755. doi: 10.1111/acel.12755 (PMC5946073; doi:10.1111/acel.12755)
Supplement: Supplementary file 2 [file ACEL-17-e12755-s002.doc]

|  | **MADT** | **1905** |
| --- | --- | --- |
| Number of individuals | 736 | 1089 |
| Average age in years at intake (range) | 50.6 (46.0–55.0) | 93.2 (92.2–93.8) |
| Males : females ratio (%) at intake | 50.4 : 49.6 | 28.7 : 71.3 |
| Mean follow up time (SD) | - | 3.53 (2.5) |
| Follow up time (years) | - | 11.4 |

Table 2S: General characteristics of Danish cohort used in this study, where MADT (Middle-Age Danish Twins) represent the controls and the 1905 Cohort the cases. N.B: Information about post-survey mortality were used in this work only for survival analysis in the oldest cohort, the Danish 1905.
