# Supplementary material for: The genetic component of human longevity: New insights from the analysis of pathway‐based SNP‐SNP interactions
Source: Aging Cell. 2018 Mar 25;17(3):e12755. doi: 10.1111/acel.12755 (PMC5946073; doi:10.1111/acel.12755)
Supplement: Supplementary file 3 [file ACEL-17-e12755-s003.docx]

| **SNP** | **VARIANT** | **CHROMOSOME** | **POSITION** | **GENE** | **PATHWAY** |
| --- | --- | --- | --- | --- | --- |
| rs14920 | A/C | 16 | 66249016 | *ACD* | DNA REPAIR |
| rs6979 | A/G | 16 | 66249169 | *ACD* | DNA REPAIR |
| rs1137582 | C/G | 17 | 71461135 | *ACOX1* | PRO/ANTIOXI |
| rs11651351 | A/G | 17 | 71478457 | *ACOX1* | PRO/ANTIOXI |
| rs12430 | A/G | 17 | 71453550 | *ACOX1* | PRO/ANTIOXI |
| rs17583163 | A/G | 17 | 71469596 | *ACOX1* | PRO/ANTIOXI |
| rs3643 | A/G | 17 | 71449260 | *ACOX1* | PRO/ANTIOXI |
| rs3744032 | A/G | 17 | 71481430 | *ACOX1* | PRO/ANTIOXI |
| rs3744033 | A/G | 17 | 71481548 | *ACOX1* | PRO/ANTIOXI |
| rs7219716 | A/C | 17 | 71468332 | *ACOX1* | PRO/ANTIOXI |
| rs7226127 | A/G | 17 | 71471061 | *ACOX1* | PRO/ANTIOXI |
| rs8065144 | A/G | 17 | 71471452 | *ACOX1* | PRO/ANTIOXI |
| rs1130214 | A/C | 14 | 104330779 | *AKT1* | IIS |
| rs2494731 | G/C | 14 | 104308725 | *AKT1* | IIS |
| rs2494732 | A/G | 14 | 104310237 | *AKT1* | IIS |
| rs2494738 | G/A | 14 | 104317731 | *AKT1* | IIS |
| rs2494748 | A/G | 14 | 104329937 | *AKT1* | IIS |
| rs2498796 | A/G | 14 | 104314265 | *AKT1* | IIS |
| rs10172572 | A/G | 2 | 201210640 | *AOX1* | PRO/ANTIOXI |
| rs10187029 | C/G | 2 | 201178036 | *AOX1* | PRO/ANTIOXI |
| rs10497866 | A/G | 2 | 201182681 | *AOX1* | PRO/ANTIOXI |
| rs11887334 | A/G | 2 | 201214198 | *AOX1* | PRO/ANTIOXI |
| rs1405984 | A/G | 2 | 201170602 | *AOX1* | PRO/ANTIOXI |
| rs16833921 | A/C | 2 | 201197710 | *AOX1* | PRO/ANTIOXI |
| rs17533245 | A/T | 2 | 201210317 | *AOX1* | PRO/ANTIOXI |
| rs2002957 | A/G | 2 | 201189757 | *AOX1* | PRO/ANTIOXI |
| rs2241080 | A/G | 2 | 201235484 | *AOX1* | PRO/ANTIOXI |
| rs2256977 | A/G | 2 | 201208428 | *AOX1* | PRO/ANTIOXI |
| rs2348021 | A/G | 2 | 201197003 | *AOX1* | PRO/ANTIOXI |
| rs2348023 | A/G | 2 | 201223550 | *AOX1* | PRO/ANTIOXI |
| rs2463489 | A/C | 2 | 201168561 | *AOX1* | PRO/ANTIOXI |
| rs2465661 | A/G | 2 | 201208876 | *AOX1* | PRO/ANTIOXI |
| rs2465665 | A/G | 2 | 201186509 | *AOX1* | PRO/ANTIOXI |
| rs2540066 | A/G | 2 | 201243320 | *AOX1* | PRO/ANTIOXI |
| rs2540069 | A/G | 2 | 201228843 | *AOX1* | PRO/ANTIOXI |
| rs2881811 | A/G | 2 | 201234991 | *AOX1* | PRO/ANTIOXI |
| rs4672866 | A/C | 2 | 201193894 | *AOX1* | PRO/ANTIOXI |
| rs6435060 | A/G | 2 | 201238145 | *AOX1* | PRO/ANTIOXI |
| rs6761375 | A/G | 2 | 201194075 | *AOX1* | PRO/ANTIOXI |
| rs7562507 | C/G | 2 | 201231429 | *AOX1* | PRO/ANTIOXI |
| rs7587871 | A/C | 2 | 201213514 | *AOX1* | PRO/ANTIOXI |
| rs3136817 | A/G | 14 | 19994274 | *APEX1* | DNA REPAIR |
| rs1016674 | A/G | 9 | 32972687 | *APTX* | DNA REPAIR |
| rs10813916 | A/G | 9 | 32980132 | *APTX* | DNA REPAIR |
| rs10971264 | A/G | 9 | 32971925 | *APTX* | DNA REPAIR |
| rs1125479 | A/C | 9 | 32973443 | *APTX* | DNA REPAIR |
| rs13296038 | G/A | 9 | 32970420 | *APTX* | DNA REPAIR |
| rs3824457 | A/G | 9 | 32974072 | *APTX* | DNA REPAIR |
| rs170548 | A/C | 11 | 107740046 | *ATM* | DNA REPAIR |
| rs17503908 | A/C | 11 | 107720607 | *ATM* | DNA REPAIR |
| rs1801516 | A/G | 11 | 107680672 | *ATM* | DNA REPAIR |
| rs611646 | A/T | 11 | 107682307 | *ATM* | DNA REPAIR |
| rs639923 | A/G | 11 | 107700103 | *ATM* | DNA REPAIR |
| rs664677 | A/G | 11 | 107648392 | *ATM* | DNA REPAIR |
| rs10804682 | A/G | 3 | 143717224 | *ATR* | DNA REPAIR |
| rs11920625 | A/G | 3 | 143744029 | *ATR* | DNA REPAIR |
| rs1802904 | A/G | 3 | 143651021 | *ATR* | DNA REPAIR |
| rs2227928 | A/G | 3 | 143764302 | *ATR* | DNA REPAIR |
| rs2229032 | G/A | 3 | 143660834 | *ATR* | DNA REPAIR |
| rs7630115 | A/G | 3 | 143693491 | *ATR* | DNA REPAIR |
| rs2270132 | A/C | 15 | 89152872 | *BLM* | DNA REPAIR |
| rs2518967 | A/G | 15 | 89112021 | *BLM* | DNA REPAIR |
| rs2518968 | C/G | 15 | 89108414 | *BLM* | DNA REPAIR |
| rs2532105 | G/A | 15 | 89156489 | *BLM* | DNA REPAIR |
| rs3784782 | G/C | 15 | 89103252 | *BLM* | DNA REPAIR |
| rs3815003 | A/G | 15 | 89113827 | *BLM* | DNA REPAIR |
| rs389480 | A/G | 15 | 89152934 | *BLM* | DNA REPAIR |
| rs401549 | A/G | 15 | 89157508 | *BLM* | DNA REPAIR |
| rs414634 | A/C | 15 | 89157257 | *BLM* | DNA REPAIR |
| rs4932365 | C/A | 15 | 89152106 | *BLM* | DNA REPAIR |
| rs7162960 | A/G | 15 | 89088416 | *BLM* | DNA REPAIR |
| rs7165790 | A/G | 15 | 89122439 | *BLM* | DNA REPAIR |
| rs7179346 | A/C | 15 | 89139164 | *BLM* | DNA REPAIR |
| rs7184015 | A/C | 15 | 89075579 | *BLM* | DNA REPAIR |
| rs16945628 | A/G | 17 | 57222011 | *BRIP1* | DNA REPAIR |
| rs16945643 | A/G | 17 | 57248772 | *BRIP1* | DNA REPAIR |
| rs2048718 | A/G | 17 | 57295601 | *BRIP1* | DNA REPAIR |
| rs2191248 | A/G | 17 | 57231134 | *BRIP1* | DNA REPAIR |
| rs2378908 | G/A | 17 | 57266547 | *BRIP1* | DNA REPAIR |
| rs4988340 | A/G | 17 | 57295415 | *BRIP1* | DNA REPAIR |
| rs6504063 | A/G | 17 | 57122283 | *BRIP1* | DNA REPAIR |
| rs8076746 | A/G | 17 | 57269499 | *BRIP1* | DNA REPAIR |
| rs8077088 | A/C | 17 | 57243200 | *BRIP1* | DNA REPAIR |
| rs9908659 | A/G | 17 | 57279644 | *BRIP1* | DNA REPAIR |
| rs3740487 | C/A | 10 | 102740773 | *C10orf2* | DNA REPAIR |
| rs12793832 | A/C | 11 | 67129169 | *c11orf72* | PRO/ANTIOXI |
| rs10488736 | A/G | 11 | 34445828 | *CAT* | PRO/ANTIOXI |
| rs11032703 | A/G | 11 | 34426232 | *CAT* | PRO/ANTIOXI |
| rs2300182 | A/T | 11 | 34424424 | *CAT* | PRO/ANTIOXI |
| rs3758730 | A/T | 11 | 34431675 | *CAT* | PRO/ANTIOXI |
| rs499406 | A/G | 11 | 34448517 | *CAT* | PRO/ANTIOXI |
| rs554518 | A/G | 11 | 34412764 | *CAT* | PRO/ANTIOXI |
| rs566979 | A/C | 11 | 34447223 | *CAT* | PRO/ANTIOXI |
| rs769214 | A/G | 11 | 34416293 | *CAT* | PRO/ANTIOXI |
| rs769217 | A/G | 11 | 34439484 | *CAT* | PRO/ANTIOXI |
| rs7933285 | A/G | 11 | 34433701 | *CAT* | PRO/ANTIOXI |
| rs13072552 | A/C | 3 | 150395816 | *CP* | PRO/ANTIOXI |
| rs13095262 | A/G | 3 | 150397852 | *CP* | PRO/ANTIOXI |
| rs17787768 | C/G | 3 | 150378221 | *CP* | PRO/ANTIOXI |
| rs3755642 | A/G | 3 | 150424291 | *CP* | PRO/ANTIOXI |
| rs3816893 | A/T | 3 | 150410401 | *CP* | PRO/ANTIOXI |
| rs4974389 | A/G | 3 | 150386613 | *CP* | PRO/ANTIOXI |
| rs701748 | A/G | 3 | 150423965 | *CP* | PRO/ANTIOXI |
| rs701753 | A/T | 3 | 150398925 | *CP* | PRO/ANTIOXI |
| rs701754 | A/T | 3 | 150387697 | *CP* | PRO/ANTIOXI |
| rs7652826 | A/G | 3 | 150421640 | *CP* | PRO/ANTIOXI |
| rs9853335 | C/G | 3 | 150394637 | *CP* | PRO/ANTIOXI |
| rs1056836 | C/G | 2 | 38151707 | *CYP1B1* | PRO/ANTIOXI |
| rs10916 | A/C | 2 | 38150674 | *CYP1B1* | PRO/ANTIOXI |
| rs162556 | A/G | 2 | 38159958 | *CYP1B1* | PRO/ANTIOXI |
| rs162557 | A/G | 2 | 38158955 | *CYP1B1* | PRO/ANTIOXI |
| rs11780874 | A/C | 8 | 145221292 | *CYC1* | PRO/ANTIOXI |
| rs10128350 | C/G | 10 | 15003678 | *DCLRE1C* | DNA REPAIR |
| rs10796227 | A/C | 10 | 15021542 | *DCLRE1C* | DNA REPAIR |
| rs11259405 | A/G | 10 | 15028606 | *DCLRE1C* | DNA REPAIR |
| rs11593133 | A/G | 10 | 15035155 | *DCLRE1C* | DNA REPAIR |
| rs12572872 | A/G | 10 | 14994030 | *DCLRE1C* | DNA REPAIR |
| rs3814176 | A/G | 10 | 15035731 | *DCLRE1C* | DNA REPAIR |
| rs7900814 | A/G | 10 | 14991667 | *DCLRE1C* | DNA REPAIR |
| rs7906952 | A/G | 10 | 15025308 | *DCLRE1C* | DNA REPAIR |
| rs7916722 | A/T | 10 | 15020687 | *DCLRE1C* | DNA REPAIR |
| rs7920514 | A/G | 10 | 15027446 | *DCLRE1C* | DNA REPAIR |
| rs17549396 | A/T | 11 | 60851923 | *DDB1* | DNA REPAIR |
| rs9651726 | A/G | 11 | 60825934 | *DDB1* | DNA REPAIR |
| rs1685404 | C/G | 11 | 47200241 | *DDB2* | DNA REPAIR |
| rs2013867 | A/G | 11 | 47216848 | *DDB2* | DNA REPAIR |
| rs2291120 | A/G | 11 | 47194256 | *DDB2* | DNA REPAIR |
| rs3824866 | G/A | 11 | 47215429 | *DDB2* | DNA REPAIR |
| rs4647709 | G/A | 11 | 47193935 | *DDB2* | DNA REPAIR |
| rs3212948 | C/G | 19 | 50616202 | *ERCC1* | DNA REPAIR |
| rs3212955 | A/G | 19 | 50615336 | *ERCC1* | DNA REPAIR |
| rs3212961 | A/C | 19 | 50614163 | *ERCC1* | DNA REPAIR |
| rs3212964 | A/G | 19 | 50612636 | *ERCC1* | DNA REPAIR |
| rs762562 | A/G | 19 | 50604183 | *ERCC1* | DNA REPAIR |
| rs1799788 | G/A | 19 | 50547940 | *ERCC2* | DNA REPAIR |
| rs238404 | A/G | 19 | 50557530 | *ERCC2* | DNA REPAIR |
| rs238407 | A/T | 19 | 50560318 | *ERCC2* | DNA REPAIR |
| rs238415 | C/G | 19 | 50549075 | *ERCC2* | DNA REPAIR |
| rs3810366 | C/G | 19 | 50565782 | *ERCC2* | DNA REPAIR |
| rs3916874 | G/C | 19 | 50548766 | *ERCC2* | DNA REPAIR |
| rs50871 | A/C | 19 | 50554355 | *ERCC2* | DNA REPAIR |
| rs50872 | A/G | 19 | 50554289 | *ERCC2* | DNA REPAIR |
| rs4150403 | G/A | 2 | 127766538 | *ERCC3* | DNA REPAIR |
| rs4150454 | A/G | 2 | 127755014 | *ERCC3* | DNA REPAIR |
| rs4150459 | G/A | 2 | 127753948 | *ERCC3* | DNA REPAIR |
| rs4150506 | A/G | 2 | 127737016 | *ERCC3* | DNA REPAIR |
| rs1800067 | G/A | 16 | 13936534 | *ERCC4* | DNA REPAIR |
| rs3136202 | G/A | 16 | 13945675 | *ERCC4* | DNA REPAIR |
| rs17655 | G/C | 13 | 102326003 | *ERCC5* | DNA REPAIR |
| rs2296147 | A/G | 13 | 102296376 | *ERCC5* | DNA REPAIR |
| rs4150350 | A/G | 13 | 102319676 | *ERCC5* | DNA REPAIR |
| rs4150351 | A/C | 13 | 102320968 | *ERCC5* | DNA REPAIR |
| rs4150355 | A/G | 13 | 102321313 | *ERCC5* | DNA REPAIR |
| rs4150386 | A/C | 13 | 102325529 | *ERCC5* | DNA REPAIR |
| rs4150393 | A/G | 13 | 102326659 | *ERCC5* | DNA REPAIR |
| rs7325708 | G/C | 13 | 102297492 | *ERCC5* | DNA REPAIR |
| rs876430 | A/G | 13 | 102327285 | *ERCC5* | DNA REPAIR |
| rs9514065 | G/A | 13 | 102292183 | *ERCC5* | DNA REPAIR |
| rs1012553 | A/G | 10 | 50410149 | *ERCC6* | DNA REPAIR |
| rs1018603 | A/C | 10 | 50398377 | *ERCC6* | DNA REPAIR |
| rs1964145 | A/C | 10 | 50411257 | *ERCC6* | DNA REPAIR |
| rs2228527 | A/G | 10 | 50348375 | *ERCC6* | DNA REPAIR |
| rs2228528 | G/A | 10 | 50402286 | *ERCC6* | DNA REPAIR |
| rs2228529 | A/G | 10 | 50337111 | *ERCC6* | DNA REPAIR |
| rs3793784 | C/G | 10 | 50417545 | *ERCC6* | DNA REPAIR |
| rs4253079 | A/C | 10 | 50392831 | *ERCC6* | DNA REPAIR |
| rs4253164 | C/A | 10 | 50361165 | *ERCC6* | DNA REPAIR |
| rs4253200 | A/G | 10 | 50350851 | *ERCC6* | DNA REPAIR |
| rs4838519 | A/C | 10 | 50340263 | *ERCC6* | DNA REPAIR |
| rs1021005 | A/G | 5 | 60227217 | *ERCC8* | DNA REPAIR |
| rs12522154 | A/G | 5 | 60217257 | *ERCC8* | DNA REPAIR |
| rs158931 | C/G | 5 | 60275455 | *ERCC8* | DNA REPAIR |
| rs158937 | A/G | 5 | 60271963 | *ERCC8* | DNA REPAIR |
| rs17332991 | C/A | 5 | 60215117 | *ERCC8* | DNA REPAIR |
| rs2306350 | A/G | 5 | 60222375 | *ERCC8* | DNA REPAIR |
| rs7726671 | A/G | 5 | 60226351 | *ERCC8* | DNA REPAIR |
| rs1047840 | A/G | 1 | 240108924 | *EXO1* | DNA REPAIR |
| rs11581448 | C/G | 1 | 240076439 | *EXO1* | DNA REPAIR |
| rs12118937 | A/G | 1 | 240075930 | *EXO1* | DNA REPAIR |
| rs12564134 | A/G | 1 | 240075450 | *EXO1* | DNA REPAIR |
| rs1635518 | A/G | 1 | 240077197 | *EXO1* | DNA REPAIR |
| rs1776131 | A/T | 1 | 240090868 | *EXO1* | DNA REPAIR |
| rs2797604 | A/G | 1 | 240094409 | *EXO1* | DNA REPAIR |
| rs4149855 | A/T | 1 | 240079229 | *EXO1* | DNA REPAIR |
| rs4149867 | G/A | 1 | 240083213 | *EXO1* | DNA REPAIR |
| rs4149965 | A/G | 1 | 240102061 | *EXO1* | DNA REPAIR |
| rs4150018 | A/C | 1 | 240119244 | *EXO1* | DNA REPAIR |
| rs4150027 | A/G | 1 | 240120461 | *EXO1* | DNA REPAIR |
| rs4408133 | C/G | 1 | 240116272 | *EXO1* | DNA REPAIR |
| rs9350 | G/A | 1 | 240115297 | *EXO1* | DNA REPAIR |
| rs1006548 | A/G | 16 | 88371544 | *FANCA* | DNA REPAIR |
| rs12924101 | A/C | 16 | 88390407 | *FANCA* | DNA REPAIR |
| rs16966142 | A/G | 16 | 88378534 | *FANCA* | DNA REPAIR |
| rs17226075 | G/A | 16 | 88389703 | *FANCA* | DNA REPAIR |
| rs1800339 | C/A | 16 | 88367138 | *FANCA* | DNA REPAIR |
| rs2238526 | A/G | 16 | 88354224 | *FANCA* | DNA REPAIR |
| rs3743860 | A/G | 16 | 88345992 | *FANCA* | DNA REPAIR |
| rs7187436 | A/T | 16 | 88372611 | *FANCA* | DNA REPAIR |
| rs7190823 | A/G | 16 | 88393544 | *FANCA* | DNA REPAIR |
| rs9282681 | A/G | 16 | 88333415 | *FANCA* | DNA REPAIR |
| rs2075310 | G/A | 3 | 10042651 | *FANCD1* | DNA REPAIR |
| rs3172417 | A/G | 3 | 10117949 | *FANCD1* | DNA REPAIR |
| rs6775725 | G/A | 3 | 10040144 | *FANCD1* | DNA REPAIR |
| rs6792811 | A/G | 3 | 10117452 | *FANCD1* | DNA REPAIR |
| rs6807485 | G/C | 3 | 10109320 | *FANCD1* | DNA REPAIR |
| rs803335 | A/C | 3 | 10060035 | *FANCD1* | DNA REPAIR |
| rs9875081 | G/A | 3 | 10058935 | *FANCD1* | DNA REPAIR |
| rs412334 | G/A | 11 | 61316837 | *FEN1* | DNA REPAIR |
| rs10507486 | G/A | 13 | 40084501 | *FOXO1* | IIS |
| rs12854161 | A/G | 13 | 40100384 | *FOXO1* | IIS |
| rs12876443 | A/G | 13 | 40094877 | *FOXO1* | IIS |
| rs2180961 | T/A | 13 | 40038044 | *FOXO1* | IIS |
| rs2701858 | G/A | 13 | 40036389 | *FOXO1* | IIS |
| rs2755209 | A/C | 13 | 40035804 | *FOXO1* | IIS |
| rs2755213 | A/G | 13 | 40044301 | *FOXO1* | IIS |
| rs2984121 | C/G | 13 | 40059979 | *FOXO1* | IIS |
| rs10499051 | A/G | 6 | 109008873 | *FOXO3* | IIS |
| rs12206094 | A/G | 6 | 109012893 | *FOXO3* | IIS |
| rs12207868 | A/G | 6 | 109071578 | *FOXO3* | IIS |
| rs12212067 | A/C | 6 | 109087889 | *FOXO3* | IIS |
| rs13217795 | A/G | 6 | 109080791 | *FOXO3* | IIS |
| rs13220810 | A/G | 6 | 109019894 | *FOXO3* | IIS |
| rs2764264 | A/G | 6 | 109041154 | *FOXO3* | IIS |
| rs2802292 | A/C | 6 | 109015211 | *FOXO3* | IIS |
| rs3800231 | G/A | 6 | 109104959 | *FOXO3* | IIS |
| rs3800232 | G/A | 6 | 109105646 | *FOXO3* | IIS |
| rs479744 | C/A | 6 | 109126725 | *FOXO3* | IIS |
| rs9398172 | A/G | 6 | 109101519 | *FOXO3* | IIS |
| rs9400239 | G/A | 6 | 109084356 | *FOXO3* | IIS |
| rs5980741 | A/C | 23 | 70231144 | *FOXO4* | IIS |
| rs16883912 | A/G | 6 | 53481730 | *GCLC* | PRO/ANTIOXI |
| rs2100375 | A/G | 6 | 53493434 | *GCLC* | PRO/ANTIOXI |
| rs2397147 | A/G | 6 | 53509546 | *GCLC* | PRO/ANTIOXI |
| rs4712035 | C/G | 6 | 53509062 | *GCLC* | PRO/ANTIOXI |
| rs531557 | A/T | 6 | 53497954 | *GCLC* | PRO/ANTIOXI |
| rs534957 | C/G | 6 | 53514310 | *GCLC* | PRO/ANTIOXI |
| rs572496 | A/G | 6 | 53485578 | *GCLC* | PRO/ANTIOXI |
| rs661603 | A/G | 6 | 53478066 | *GCLC* | PRO/ANTIOXI |
| rs670548 | A/G | 6 | 53474948 | *GCLC* | PRO/ANTIOXI |
| rs675908 | A/G | 6 | 53521259 | *GCLC* | PRO/ANTIOXI |
| rs7742367 | A/G | 6 | 53469235 | *GCLC* | PRO/ANTIOXI |
| rs2854184 | A/T | 17 | 59351725 | *GH1* | IIS |
| rs3020619 | A/G | 17 | 59346869 | *GH1* | IIS |
| rs11739840 | A/G | 5 | 42714102 | *GHR* | IIS |
| rs11744988 | G/A | 5 | 42510453 | *GHR* | IIS |
| rs12153009 | A/G | 5 | 42589636 | *GHR* | IIS |
| rs12187996 | A/G | 5 | 42753367 | *GHR* | IIS |
| rs12233949 | C/G | 5 | 42643218 | *GHR* | IIS |
| rs13182117 | A/C | 5 | 42647605 | *GHR* | IIS |
| rs1509453 | A/G | 5 | 42531092 | *GHR* | IIS |
| rs17230998 | A/C | 5 | 42750449 | *GHR* | IIS |
| rs17574650 | A/C | 5 | 42472673 | *GHR* | IIS |
| rs2940918 | A/C | 5 | 42506342 | *GHR* | IIS |
| rs2972418 | A/G | 5 | 42519450 | *GHR* | IIS |
| rs4130113 | A/G | 5 | 42550408 | *GHR* | IIS |
| rs4410646 | A/C | 5 | 42605399 | *GHR* | IIS |
| rs4547964 | A/G | 5 | 42715853 | *GHR* | IIS |
| rs6180 | A/C | 5 | 42754996 | *GHR* | IIS |
| rs6451620 | G/A | 5 | 42508871 | *GHR* | IIS |
| rs6451634 | G/A | 5 | 42720694 | *GHR* | IIS |
| rs6883523 | A/C | 5 | 42600505 | *GHR* | IIS |
| rs7702524 | A/G | 5 | 42649655 | *GHR* | IIS |
| rs7703713 | A/G | 5 | 42591688 | *GHR* | IIS |
| rs1073768 | A/G | 20 | 35310424 | *GHRH* | IIS |
| rs6032470 | A/G | 20 | 35320941 | *GHRH* | IIS |
| rs10225302 | A/G | 7 | 30967600 | *GHRHR* | IIS |
| rs11761979 | A/C | 7 | 30984663 | *GHRHR* | IIS |
| rs2267723 | A/G | 7 | 30973467 | *GHRHR* | IIS |
| rs4988496 | G/A | 7 | 30975211 | *GHRHR* | IIS |
| rs4988498 | C/A | 7 | 30976101 | *GHRHR* | IIS |
| rs4988501 | A/G | 7 | 30978010 | *GHRHR* | IIS |
| rs4988504 | A/G | 7 | 30982543 | *GHRHR* | IIS |
| rs4988505 | C/G | 7 | 30983628 | *GHRHR* | IIS |
| rs6954044 | T/A | 7 | 30968540 | *GHRHR* | IIS |
| rs10490815 | A/G | 3 | 10310145 | *GHRL* | IIS |
| rs1617161 | G/A | 3 | 10311853 | *GHRL* | IIS |
| rs26802 | A/C | 3 | 10307365 | *GHRL* | IIS |
| rs27498 | A/G | 3 | 10309021 | *GHRL* | IIS |
| rs27647 | A/G | 3 | 10307468 | *GHRL* | IIS |
| rs35682 | A/G | 3 | 10303782 | *GHRL* | IIS |
| rs3755777 | C/G | 3 | 10308364 | *GHRL* | IIS |
| rs42451 | A/G | 3 | 10305377 | *GHRL* | IIS |
| rs696217 | C/A | 3 | 10306457 | *GHRL* | IIS |
| rs512692 | A/T | 3 | 173653536 | *GHSR* | IIS |
| rs572169 | A/G | 3 | 173648421 | *GHSR* | IIS |
| rs9819506 | A/G | 3 | 173652798 | *GHSR* | IIS |
| rs1047420 | A/G | 5 | 95176001 | *GLRX* | PRO/ANTIOXI |
| rs12513728 | A/C | 5 | 95187501 | *GLRX* | PRO/ANTIOXI |
| rs2007 | C/G | 5 | 95175411 | *GLRX* | PRO/ANTIOXI |
| rs3756704 | A/G | 5 | 95186050 | *GLRX* | PRO/ANTIOXI |
| rs4561 | A/G | 5 | 95178069 | *GLRX* | PRO/ANTIOXI |
| rs871775 | A/G | 5 | 95184524 | *GLRX* | PRO/ANTIOXI |
| rs9314160 | A/G | 5 | 95182213 | *GLRX* | PRO/ANTIOXI |
| rs1946234 | A/C | 5 | 150379403 | *GPX3* | PRO/ANTIOXI |
| rs2042235 | A/G | 5 | 150378107 | *GPX3* | PRO/ANTIOXI |
| rs2042236 | A/G | 5 | 150378308 | *GPX3* | PRO/ANTIOXI |
| rs3792798 | A/G | 5 | 150381958 | *GPX3* | PRO/ANTIOXI |
| rs3805435 | A/G | 5 | 150381489 | *GPX3* | PRO/ANTIOXI |
| rs4958434 | A/G | 5 | 150382763 | *GPX3* | PRO/ANTIOXI |
| rs4958872 | A/G | 5 | 150382527 | *GPX3* | PRO/ANTIOXI |
| rs8177431 | A/G | 5 | 150384189 | *GPX3* | PRO/ANTIOXI |
| rs870407 | A/G | 5 | 150380780 | *GPX3* | PRO/ANTIOXI |
| rs3746165 | A/G | 19 | 1053211 | *GPX4* | PRO/ANTIOXI |
| rs4588110 | A/G | 19 | 1053139 | *GPX4* | PRO/ANTIOXI |
| rs4807542 | A/G | 19 | 1055078 | *GPX4* | PRO/ANTIOXI |
| rs8178977 | C/G | 19 | 1057477 | *GPX4* | PRO/ANTIOXI |
| rs1002149 | A/C | 8 | 30705280 | *GSR* | PRO/ANTIOXI |
| rs17557435 | A/G | 8 | 30695380 | *GSR* | PRO/ANTIOXI |
| rs2253409 | C/G | 8 | 30666508 | *GSR* | PRO/ANTIOXI |
| rs3594 | A/C | 8 | 30655202 | *GSR* | PRO/ANTIOXI |
| rs3779647 | A/G | 8 | 30680429 | *GSR* | PRO/ANTIOXI |
| rs8190996 | A/G | 8 | 30673548 | *GSR* | PRO/ANTIOXI |
| rs17309872 | A/T | 20 | 32979449 | *GSS* | PRO/ANTIOXI |
| rs17310467 | A/G | 20 | 33009277 | *GSS* | PRO/ANTIOXI |
| rs2236270 | A/C | 20 | 32986816 | *GSS* | PRO/ANTIOXI |
| rs6088660 | A/G | 20 | 33006557 | *GSS* | PRO/ANTIOXI |
| rs6088662 | A/C | 20 | 33011294 | *GSS* | PRO/ANTIOXI |
| rs725521 | A/G | 20 | 32979732 | *GSS* | PRO/ANTIOXI |
| rs10735234 | A/G | 1 | 110083464 | *GSTM3* | PRO/ANTIOXI |
| rs7483 | A/G | 1 | 110081224 | *GSTM3* | PRO/ANTIOXI |
| rs1138272 | A/G | 11 | 67110155 | *GSTP1* | PRO/ANTIOXI |
| rs1695 | A/G | 11 | 67109265 | *GSTP1* | PRO/ANTIOXI |
| rs7927381 | A/G | 11 | 67103319 | *GSTP1* | PRO/ANTIOXI |
| rs7941395 | A/G | 11 | 67103993 | *GSTP1* | PRO/ANTIOXI |
| rs2509049 | A/G | 11 | 118471731 | *H2AFX* | DNA REPAIR |
| rs640603 | A/G | 11 | 118469540 | *H2AFX* | DNA REPAIR |
| rs11187033 | A/T | 10 | 94252339 | *IDE* | IIS |
| rs11187065 | A/G | 10 | 94301904 | *IDE* | IIS |
| rs17445328 | A/G | 10 | 94295169 | *IDE* | IIS |
| rs1887922 | A/G | 10 | 94214145 | *IDE* | IIS |
| rs2275218 | A/G | 10 | 94215277 | *IDE* | IIS |
| rs2421943 | A/G | 10 | 94301795 | *IDE* | IIS |
| rs4646957 | A/G | 10 | 94219892 | *IDE* | IIS |
| rs6583820 | A/G | 10 | 94266635 | *IDE* | IIS |
| rs7078413 | A/C | 10 | 94280464 | *IDE* | IIS |
| rs7899603 | C/G | 10 | 94214997 | *IDE* | IIS |
| rs1019731 | C/A | 12 | 101388555 | *IGF1* | IIS |
| rs10735380 | A/G | 12 | 101368366 | *IGF1* | IIS |
| rs10778176 | A/G | 12 | 101387109 | *IGF1* | IIS |
| rs10860865 | C/A | 12 | 101355852 | *IGF1* | IIS |
| rs11111262 | G/A | 12 | 101322307 | *IGF1* | IIS |
| rs12821878 | A/G | 12 | 101391797 | *IGF1* | IIS |
| rs1520220 | C/G | 12 | 101320652 | *IGF1* | IIS |
| rs17727841 | C/G | 12 | 101333760 | *IGF1* | IIS |
| rs5742632 | A/G | 12 | 101380604 | *IGF1* | IIS |
| rs6214 | A/G | 12 | 101317699 | *IGF1* | IIS |
| rs11247367 | G/A | 15 | 97053799 | *IGF1R* | IIS |
| rs11630259 | A/G | 15 | 97248471 | *IGF1R* | IIS |
| rs11630479 | A/G | 15 | 97058004 | *IGF1R* | IIS |
| rs12437963 | A/G | 15 | 97314382 | *IGF1R* | IIS |
| rs12440962 | A/G | 15 | 97317587 | *IGF1R* | IIS |
| rs12442093 | A/G | 15 | 97283409 | *IGF1R* | IIS |
| rs12592205 | A/T | 15 | 97308119 | *IGF1R* | IIS |
| rs12910200 | A/T | 15 | 97262992 | *IGF1R* | IIS |
| rs12916884 | A/C | 15 | 97305411 | *IGF1R* | IIS |
| rs1546713 | A/G | 15 | 97289389 | *IGF1R* | IIS |
| rs1815009 | A/G | 15 | 97322194 | *IGF1R* | IIS |
| rs1879613 | A/G | 15 | 97251880 | *IGF1R* | IIS |
| rs2017500 | A/G | 15 | 97013635 | *IGF1R* | IIS |
| rs2139924 | A/C | 15 | 97272793 | *IGF1R* | IIS |
| rs2684787 | G/A | 15 | 97322585 | *IGF1R* | IIS |
| rs2684788 | A/G | 15 | 97321960 | *IGF1R* | IIS |
| rs2684790 | G/A | 15 | 97309063 | *IGF1R* | IIS |
| rs2684792 | A/G | 15 | 97305604 | *IGF1R* | IIS |
| rs2684796 | G/A | 15 | 97300915 | *IGF1R* | IIS |
| rs2684799 | G/A | 15 | 97299355 | *IGF1R* | IIS |
| rs2684802 | A/C | 15 | 97289030 | *IGF1R* | IIS |
| rs2684806 | A/G | 15 | 97282808 | *IGF1R* | IIS |
| rs2684807 | A/G | 15 | 97281977 | *IGF1R* | IIS |
| rs2684811 | A/G | 15 | 97271086 | *IGF1R* | IIS |
| rs2715416 | C/G | 15 | 97271554 | *IGF1R* | IIS |
| rs2715419 | A/G | 15 | 97302243 | *IGF1R* | IIS |
| rs3743254 | G/A | 15 | 97317453 | *IGF1R* | IIS |
| rs3743258 | A/G | 15 | 97260662 | *IGF1R* | IIS |
| rs3784605 | A/G | 15 | 97275850 | *IGF1R* | IIS |
| rs4965438 | A/C | 15 | 97288952 | *IGF1R* | IIS |
| rs4966008 | A/G | 15 | 97020968 | *IGF1R* | IIS |
| rs4966013 | A/G | 15 | 97062907 | *IGF1R* | IIS |
| rs4966038 | C/G | 15 | 97264155 | *IGF1R* | IIS |
| rs4966039 | A/G | 15 | 97275338 | *IGF1R* | IIS |
| rs4966042 | A/G | 15 | 97281626 | *IGF1R* | IIS |
| rs4966046 | A/G | 15 | 97292798 | *IGF1R* | IIS |
| rs7162336 | G/A | 15 | 97293049 | *IGF1R* | IIS |
| rs7166348 | G/A | 15 | 97065318 | *IGF1R* | IIS |
| rs7168671 | A/G | 15 | 97272485 | *IGF1R* | IIS |
| rs7173191 | A/G | 15 | 97044958 | *IGF1R* | IIS |
| rs8026157 | A/G | 15 | 97313910 | *IGF1R* | IIS |
| rs8032477 | A/G | 15 | 97072077 | *IGF1R* | IIS |
| rs8038056 | A/G | 15 | 97300451 | *IGF1R* | IIS |
| rs867431 | A/G | 15 | 97267206 | *IGF1R* | IIS |
| rs871335 | C/A | 15 | 97297469 | *IGF1R* | IIS |
| rs939626 | A/G | 15 | 97310699 | *IGF1R* | IIS |
| rs9672254 | A/G | 15 | 97315608 | *IGF1R* | IIS |
| rs9920651 | A/G | 15 | 97280163 | *IGF1R* | IIS |
| rs1004446 | A/G | 11 | 2126719 | *IGF2* | IIS |
| rs10770125 | A/G | 11 | 2125590 | *IGF2* | IIS |
| rs17885652 | A/G | 11 | 2123879 | *IGF2* | IIS |
| rs2239681 | A/G | 11 | 2124557 | *IGF2* | IIS |
| rs2585 | A/G | 11 | 2107020 | *IGF2* | IIS |
| rs3213216 | A/G | 11 | 2114755 | *IGF2* | IIS |
| rs3741211 | A/G | 11 | 2125686 | *IGF2* | IIS |
| rs734351 | A/G | 11 | 2112789 | *IGF2* | IIS |
| rs7481173 | A/C | 11 | 2120429 | *IGF2* | IIS |
| rs11705701 | A/G | 3 | 187027003 | *IGF2BP2* | IIS |
| rs11708719 | A/G | 3 | 186997968 | *IGF2BP2* | IIS |
| rs12635769 | A/G | 3 | 186929732 | *IGF2BP2* | IIS |
| rs1447890 | C/G | 3 | 186927255 | *IGF2BP2* | IIS |
| rs17293846 | A/G | 3 | 186903006 | *IGF2BP2* | IIS |
| rs2290066 | A/G | 3 | 186928536 | *IGF2BP2* | IIS |
| rs4402960 | A/C | 3 | 186994381 | *IGF2BP2* | IIS |
| rs4686692 | A/G | 3 | 186963762 | *IGF2BP2* | IIS |
| rs6770227 | C/G | 3 | 186891464 | *IGF2BP2* | IIS |
| rs6778126 | A/G | 3 | 186888475 | *IGF2BP2* | IIS |
| rs6794209 | A/G | 3 | 186928454 | *IGF2BP2* | IIS |
| rs6799330 | A/C | 3 | 186961651 | *IGF2BP2* | IIS |
| rs7634540 | A/T | 3 | 186994182 | *IGF2BP2* | IIS |
| rs7648605 | A/C | 3 | 186973428 | *IGF2BP2* | IIS |
| rs1003737 | A/C | 6 | 160350646 | *IGF2R* | IIS |
| rs10945649 | A/T | 6 | 160348551 | *IGF2R* | IIS |
| rs2297370 | T/A | 6 | 160444763 | *IGF2R* | IIS |
| rs3734181 | A/G | 6 | 160349873 | *IGF2R* | IIS |
| rs3798186 | A/G | 6 | 160367007 | *IGF2R* | IIS |
| rs3798187 | A/G | 6 | 160356193 | *IGF2R* | IIS |
| rs3798189 | A/C | 6 | 160355559 | *IGF2R* | IIS |
| rs3798207 | A/G | 6 | 160314237 | *IGF2R* | IIS |
| rs4709395 | A/G | 6 | 160398779 | *IGF2R* | IIS |
| rs633863 | A/G | 6 | 160310773 | *IGF2R* | IIS |
| rs6917747 | G/A | 6 | 160322695 | *IGF2R* | IIS |
| rs7755435 | A/G | 6 | 160340496 | *IGF2R* | IIS |
| rs8191745 | A/T | 6 | 160352321 | *IGF2R* | IIS |
| rs8191772 | A/G | 6 | 160381324 | *IGF2R* | IIS |
| rs8191829 | A/G | 6 | 160399468 | *IGF2R* | IIS |
| rs9295119 | A/G | 6 | 160342775 | *IGF2R* | IIS |
| rs9347380 | A/G | 6 | 160357337 | *IGF2R* | IIS |
| rs9456490 | G/A | 6 | 160318320 | *IGF2R* | IIS |
| rs9457795 | A/G | 6 | 160315622 | *IGF2R* | IIS |
| rs344352 | G/C | 16 | 1786477 | *IGFALS* | IIS |
| rs1525608 | A/G | 2 | 217202000 | *IGFBP2* | IIS |
| rs2270360 | A/C | 2 | 217233231 | *IGFBP2* | IIS |
| rs3770473 | A/C | 2 | 217207084 | *IGFBP2* | IIS |
| rs6413492 | T/A | 2 | 217237196 | *IGFBP2* | IIS |
| rs9341105 | A/G | 2 | 217209064 | *IGFBP2* | IIS |
| rs9341130 | A/G | 2 | 217214510 | *IGFBP2* | IIS |
| rs9341134 | A/T | 2 | 217216171 | *IGFBP2* | IIS |
| rs9341191 | A/G | 2 | 217229893 | *IGFBP2* | IIS |
| rs13223993 | A/G | 7 | 45917755 | *IGFBP3* | IIS |
| rs2132571 | G/A | 7 | 45928199 | *IGFBP3* | IIS |
| rs2453836 | A/G | 7 | 45932320 | *IGFBP3* | IIS |
| rs2453839 | A/G | 7 | 45920098 | *IGFBP3* | IIS |
| rs2471551 | G/C | 7 | 45923580 | *IGFBP3* | IIS |
| rs3110697 | A/G | 7 | 45921554 | *IGFBP3* | IIS |
| rs6670 | A/T | 7 | 45918779 | *IGFBP3* | IIS |
| rs924140 | A/G | 7 | 45929639 | *IGFBP3* | IIS |
| rs9282734 | A/C | 7 | 45923494 | *IGFBP3* | IIS |
| rs3842748 | C/G | 11 | 2137971 | *INS* | IIS |
| rs1035939 | A/G | 19 | 7139979 | *INSR* | IIS |
| rs10401628 | G/A | 19 | 7077218 | *INSR* | IIS |
| rs11667110 | G/C | 19 | 7087609 | *INSR* | IIS |
| rs11672739 | A/G | 19 | 7085266 | *INSR* | IIS |
| rs11880337 | A/G | 19 | 7247452 | *INSR* | IIS |
| rs12979424 | A/G | 19 | 7224492 | *INSR* | IIS |
| rs1549616 | G/A | 19 | 7083570 | *INSR* | IIS |
| rs1799817 | A/G | 19 | 7076297 | *INSR* | IIS |
| rs2059807 | A/G | 19 | 7117109 | *INSR* | IIS |
| rs2252673 | C/G | 19 | 7101418 | *INSR* | IIS |
| rs2860175 | A/G | 19 | 7083081 | *INSR* | IIS |
| rs2860183 | A/G | 19 | 7140375 | *INSR* | IIS |
| rs2860184 | C/G | 19 | 7238748 | *INSR* | IIS |
| rs2963 | A/G | 19 | 7114154 | *INSR* | IIS |
| rs6510949 | C/A | 19 | 7085402 | *INSR* | IIS |
| rs6510956 | A/C | 19 | 7120276 | *INSR* | IIS |
| rs6510959 | G/A | 19 | 7135238 | *INSR* | IIS |
| rs7254060 | G/A | 19 | 7234414 | *INSR* | IIS |
| rs7254487 | A/G | 19 | 7220321 | *INSR* | IIS |
| rs7254921 | A/G | 19 | 7229452 | *INSR* | IIS |
| rs8103483 | A/G | 19 | 7096374 | *INSR* | IIS |
| rs8110428 | G/A | 19 | 7122662 | *INSR* | IIS |
| rs8111710 | C/A | 19 | 7243583 | *INSR* | IIS |
| rs8112883 | C/A | 19 | 7130320 | *INSR* | IIS |
| rs891087 | A/G | 19 | 7135518 | *INSR* | IIS |
| rs1025333 | T/A | 2 | 227353769 | *IRS1* | IIS |
| rs1560251 | C/A | 2 | 227362192 | *IRS1* | IIS |
| rs17208239 | C/A | 2 | 227305941 | *IRS1* | IIS |
| rs17208470 | C/A | 2 | 227312149 | *IRS1* | IIS |
| rs17508368 | G/A | 2 | 227359690 | *IRS1* | IIS |
| rs6725330 | A/G | 2 | 227375101 | *IRS1* | IIS |
| rs9282766 | G/A | 2 | 227363592 | *IRS1* | IIS |
| rs11618950 | A/G | 13 | 109232311 | *IRS2* | IIS |
| rs12584136 | C/A | 13 | 109217355 | *IRS2* | IIS |
| rs2099435 | A/G | 13 | 109207906 | *IRS2* | IIS |
| rs4773094 | A/G | 13 | 109239484 | *IRS2* | IIS |
| rs7323191 | A/T | 13 | 109222076 | *IRS2* | IIS |
| rs754204 | A/G | 13 | 109209569 | *IRS2* | IIS |
| rs7997595 | G/C | 13 | 109228769 | *IRS2* | IIS |
| rs7999797 | A/G | 13 | 109224001 | *IRS2* | IIS |
| rs913949 | A/G | 13 | 109209797 | *IRS2* | IIS |
| rs9515119 | A/C | 13 | 109207337 | *IRS2* | IIS |
| rs9521512 | A/G | 13 | 109239599 | *IRS2* | IIS |
| rs9559648 | A/G | 13 | 109221796 | *IRS2* | IIS |
| rs9559656 | C/G | 13 | 109227445 | *IRS2* | IIS |
| rs1888057 | A/G | 13 | 32520695 | *KL* | IIS |
| rs2283368 | A/G | 13 | 32491270 | *KL* | IIS |
| rs2320762 | A/C | 13 | 32515174 | *KL* | IIS |
| rs385564 | C/G | 13 | 32490409 | *KL* | IIS |
| rs397703 | A/G | 13 | 32485329 | *KL* | IIS |
| rs495392 | A/C | 13 | 32490193 | *KL* | IIS |
| rs522796 | A/G | 13 | 32528055 | *KL* | IIS |
| rs562020 | A/G | 13 | 32490070 | *KL* | IIS |
| rs564481 | A/G | 13 | 32532983 | *KL* | IIS |
| rs575536 | A/G | 13 | 32490777 | *KL* | IIS |
| rs576404 | A/C | 13 | 32491100 | *KL* | IIS |
| rs648202 | A/G | 13 | 32533463 | *KL* | IIS |
| rs657049 | A/G | 13 | 32520817 | *KL* | IIS |
| rs687045 | A/C | 13 | 32522889 | *KL* | IIS |
| rs9526984 | A/G | 13 | 32507937 | *KL* | IIS |
| rs9527026 | A/G | 13 | 32526239 | *KL* | IIS |
| rs9536314 | A/C | 13 | 32526138 | *KL* | IIS |
| rs156640 | C/G | 19 | 53322575 | *LIG1* | DNA REPAIR |
| rs156641 | A/G | 19 | 53323220 | *LIG1* | DNA REPAIR |
| rs3731037 | G/A | 19 | 53315036 | *LIG1* | DNA REPAIR |
| rs7246512 | G/A | 19 | 53343814 | *LIG1* | DNA REPAIR |
| rs8100261 | A/G | 19 | 53347571 | *LIG1* | DNA REPAIR |
| rs1052536 | A/G | 17 | 30355688 | *LIG3* | DNA REPAIR |
| rs3135998 | A/G | 17 | 30346435 | *LIG3* | DNA REPAIR |
| rs10131 | A/G | 13 | 107657847 | *LIG4* | DNA REPAIR |
| rs1151402 | A/G | 13 | 107656031 | *LIG4* | DNA REPAIR |
| rs1805386 | A/G | 13 | 107659914 | *LIG4* | DNA REPAIR |
| rs1805388 | A/G | 13 | 107661592 | *LIG4* | DNA REPAIR |
| rs3093748 | A/G | 13 | 107663462 | *LIG4* | DNA REPAIR |
| rs2436508 | A/G | 19 | 5662217 | *LONP1* | DNA REPAIR |
| rs2436509 | A/G | 19 | 5656647 | *LONP1* | DNA REPAIR |
| rs2436514 | A/G | 19 | 5645630 | *LONP1* | DNA REPAIR |
| rs3818815 | G/C | 19 | 5643311 | *LONP1* | DNA REPAIR |
| rs3892355 | A/G | 19 | 5647962 | *LONP1* | DNA REPAIR |
| rs17352686 | A/C | 5 | 121446444 | *LOX* | PRO/ANTIOXI |
| rs1540354 | T/A | 3 | 37019493 | *MLH1* | DNA REPAIR |
| rs1799977 | A/G | 3 | 37028572 | *MLH1* | DNA REPAIR |
| rs1800734 | A/G | 3 | 37009950 | *MLH1* | DNA REPAIR |
| rs3774335 | A/C | 3 | 37047631 | *MLH1* | DNA REPAIR |
| rs175076 | A/G | 14 | 74575239 | *MLH3* | DNA REPAIR |
| rs175080 | A/G | 14 | 74583581 | *MLH3* | DNA REPAIR |
| rs7156586 | A/T | 14 | 74575983 | *MLH3* | DNA REPAIR |
| rs10831227 | A/G | 11 | 93795225 | *MRE11A* | DNA REPAIR |
| rs13447717 | A/G | 11 | 93809099 | *MRE11A* | DNA REPAIR |
| rs13447720 | A/G | 11 | 93804974 | *MRE11A* | DNA REPAIR |
| rs16920467 | A/T | 11 | 93827574 | *MRE11A* | DNA REPAIR |
| rs1805363 | G/A | 11 | 93866600 | *MRE11A* | DNA REPAIR |
| rs500558 | A/G | 11 | 93810322 | *MRE11A* | DNA REPAIR |
| rs512150 | A/T | 11 | 93860853 | *MRE11A* | DNA REPAIR |
| rs533984 | A/G | 11 | 93838920 | *MRE11A* | DNA REPAIR |
| rs569143 | C/G | 11 | 93828035 | *MRE11A* | DNA REPAIR |
| rs592068 | A/G | 11 | 93826603 | *MRE11A* | DNA REPAIR |
| rs604845 | A/G | 11 | 93822337 | *MRE11A* | DNA REPAIR |
| rs680695 | A/G | 11 | 93851802 | *MRE11A* | DNA REPAIR |
| rs12999145 | A/G | 2 | 47540190 | *MSH2* | DNA REPAIR |
| rs17036577 | A/G | 2 | 47520342 | *MSH2* | DNA REPAIR |
| rs1863332 | A/C | 2 | 47483402 | *MSH2* | DNA REPAIR |
| rs1981929 | A/G | 2 | 47526073 | *MSH2* | DNA REPAIR |
| rs2347794 | A/G | 2 | 47510305 | *MSH2* | DNA REPAIR |
| rs3732183 | G/A | 2 | 47547463 | *MSH2* | DNA REPAIR |
| rs4638843 | G/C | 2 | 47557531 | *MSH2* | DNA REPAIR |
| rs4952887 | G/A | 2 | 47500472 | *MSH2* | DNA REPAIR |
| rs6741393 | G/A | 2 | 47543424 | *MSH2* | DNA REPAIR |
| rs6753135 | G/A | 2 | 47511188 | *MSH2* | DNA REPAIR |
| rs1650737 | A/G | 5 | 80037541 | *MSH3* | DNA REPAIR |
| rs1677645 | A/G | 5 | 80000175 | *MSH3* | DNA REPAIR |
| rs17206221 | C/G | 5 | 80062275 | *MSH3* | DNA REPAIR |
| rs181747 | A/G | 5 | 80052630 | *MSH3* | DNA REPAIR |
| rs1979005 | G/A | 5 | 80185458 | *MSH3* | DNA REPAIR |
| rs245011 | A/G | 5 | 80085382 | *MSH3* | DNA REPAIR |
| rs245378 | G/A | 5 | 80167789 | *MSH3* | DNA REPAIR |
| rs249633 | A/G | 5 | 80161909 | *MSH3* | DNA REPAIR |
| rs26279 | A/G | 5 | 80204693 | *MSH3* | DNA REPAIR |
| rs26784 | A/G | 5 | 80085648 | *MSH3* | DNA REPAIR |
| rs27385 | A/G | 5 | 80205428 | *MSH3* | DNA REPAIR |
| rs397628 | A/G | 5 | 80101891 | *MSH3* | DNA REPAIR |
| rs40139 | A/G | 5 | 80069591 | *MSH3* | DNA REPAIR |
| rs6151616 | A/G | 5 | 79997226 | *MSH3* | DNA REPAIR |
| rs6151627 | A/G | 5 | 80001292 | *MSH3* | DNA REPAIR |
| rs6151735 | G/A | 5 | 80061227 | *MSH3* | DNA REPAIR |
| rs6151792 | G/A | 5 | 80092717 | *MSH3* | DNA REPAIR |
| rs6151816 | G/A | 5 | 80109390 | *MSH3* | DNA REPAIR |
| rs7712332 | A/G | 5 | 79993591 | *MSH3* | DNA REPAIR |
| rs2020911 | A/T | 2 | 47884342 | *MSH6* | DNA REPAIR |
| rs2348244 | A/G | 2 | 47872989 | *MSH6* | DNA REPAIR |
| rs3136228 | A/C | 2 | 47863320 | *MSH6* | DNA REPAIR |
| rs3136245 | G/A | 2 | 47866350 | *MSH6* | DNA REPAIR |
| rs3136284 | A/G | 2 | 47872160 | *MSH6* | DNA REPAIR |
| rs3136329 | A/G | 2 | 47878380 | *MSH6* | DNA REPAIR |
| rs330792 | A/C | 2 | 47862078 | *MSH6* | DNA REPAIR |
| rs1001362 | A/G | 16 | 55232359 | *MT1A* | PRO/ANTIOXI |
| rs4784701 | A/C | 16 | 55228168 | *MT1A* | PRO/ANTIOXI |
| rs12680687 | A/C | 8 | 91020564 | *NBN* | DNA REPAIR |
| rs1805794 | G/C | 8 | 91059655 | *NBN* | DNA REPAIR |
| rs2735385 | A/C | 8 | 91018900 | *NBN* | DNA REPAIR |
| rs3026271 | A/G | 8 | 91052428 | *NBN* | DNA REPAIR |
| rs6470522 | G/A | 8 | 91023657 | *NBN* | DNA REPAIR |
| rs6999227 | C/G | 8 | 91022515 | *NBN* | DNA REPAIR |
| rs7010210 | A/C | 8 | 91039197 | *NBN* | DNA REPAIR |
| rs4147719 | A/G | 2 | 206714921 | *NDUFS1* | PRO/ANTIOXI |
| rs6435326 | A/T | 2 | 206710804 | *NDUFS1* | PRO/ANTIOXI |
| rs1039825 | A/C | 18 | 9104096 | *NDUFV2* | PRO/ANTIOXI |
| rs11872481 | A/G | 18 | 9118453 | *NDUFV2* | PRO/ANTIOXI |
| rs17413344 | A/G | 18 | 9110061 | *NDUFV2* | PRO/ANTIOXI |
| rs4148965 | A/C | 18 | 9099484 | *NDUFV2* | PRO/ANTIOXI |
| rs4148966 | A/C | 18 | 9110265 | *NDUFV2* | PRO/ANTIOXI |
| rs906807 | A/G | 18 | 9107867 | *NDUFV2* | PRO/ANTIOXI |
| rs977581 | A/C | 18 | 9109035 | *NDUFV2* | PRO/ANTIOXI |
| rs1043180 | A/G | 8 | 11682230 | *NEIL2* | DNA REPAIR |
| rs1466785 | G/A | 8 | 11660865 | *NEIL2* | DNA REPAIR |
| rs1874546 | C/G | 8 | 11676287 | *NEIL2* | DNA REPAIR |
| rs2686211 | A/T | 8 | 11665784 | *NEIL2* | DNA REPAIR |
| rs4840583 | A/G | 8 | 11673355 | *NEIL2* | DNA REPAIR |
| rs4841593 | C/G | 8 | 11663270 | *NEIL2* | DNA REPAIR |
| rs804269 | A/G | 8 | 11666528 | *NEIL2* | DNA REPAIR |
| rs8191534 | A/T | 8 | 11666776 | *NEIL2* | DNA REPAIR |
| rs8191542 | G/C | 8 | 11667218 | *NEIL2* | DNA REPAIR |
| rs8191589 | T/A | 8 | 11671603 | *NEIL2* | DNA REPAIR |
| rs8191605 | C/A | 8 | 11674311 | *NEIL2* | DNA REPAIR |
| rs8191663 | A/G | 8 | 11680868 | *NEIL2* | DNA REPAIR |
| rs904009 | A/C | 8 | 11665538 | *NEIL2* | DNA REPAIR |
| rs12703107 | A/C | 7 | 150314562 | *NOS3* | PRO/ANTIOXI |
| rs1541861 | A/C | 7 | 150328266 | *NOS3* | PRO/ANTIOXI |
| rs1799983 | A/C | 7 | 150327044 | *NOS3* | PRO/ANTIOXI |
| rs1800779 | A/G | 7 | 150320876 | *NOS3* | PRO/ANTIOXI |
| rs1808593 | A/C | 7 | 150339235 | *NOS3* | PRO/ANTIOXI |
| rs2853792 | A/G | 7 | 150330810 | *NOS3* | PRO/ANTIOXI |
| rs3793342 | A/G | 7 | 150326128 | *NOS3* | PRO/ANTIOXI |
| rs3918186 | A/T | 7 | 150333365 | *NOS3* | PRO/ANTIOXI |
| rs3918188 | A/C | 7 | 150333714 | *NOS3* | PRO/ANTIOXI |
| rs3918227 | A/C | 7 | 150331879 | *NOS3* | PRO/ANTIOXI |
| rs7830 | A/C | 7 | 150340504 | *NOS3* | PRO/ANTIOXI |
| rs10126566 | A/G | 23 | 100020918 | *NOX1* | PRO/ANTIOXI |
| rs12156942 | A/G | 23 | 100020232 | *NOX1* | PRO/ANTIOXI |
| rs4828068 | A/G | 23 | 99996030 | *NOX1* | PRO/ANTIOXI |
| rs4828071 | A/G | 23 | 100015595 | *NOX1* | PRO/ANTIOXI |
| rs5921669 | A/G | 23 | 99992074 | *NOX1* | PRO/ANTIOXI |
| rs5921682 | A/G | 23 | 100017093 | *NOX1* | PRO/ANTIOXI |
| rs6620949 | C/G | 23 | 100018240 | *NOX1* | PRO/ANTIOXI |
| rs2516739 | A/G | 16 | 2037159 | *NTHL1* | DNA REPAIR |
| rs17252807 | A/G | 3 | 9778126 | *OGG1* | DNA REPAIR |
| rs2304277 | A/G | 3 | 9776080 | *OGG1* | DNA REPAIR |
| rs2472037 | A/G | 3 | 9770372 | *OGG1* | DNA REPAIR |
| rs293796 | A/G | 3 | 9784082 | *OGG1* | DNA REPAIR |
| rs3218997 | G/A | 3 | 9766344 | *OGG1* | DNA REPAIR |
| rs10122701 | A/G | 9 | 118066783 | *PAPPA* | IIS |
| rs10817866 | A/G | 9 | 118066897 | *PAPPA* | IIS |
| rs12236532 | A/G | 9 | 118188490 | *PAPPA* | IIS |
| rs12344396 | C/G | 9 | 117961148 | *PAPPA* | IIS |
| rs1998499 | A/C | 9 | 118115763 | *PAPPA* | IIS |
| rs3761843 | A/G | 9 | 118136010 | *PAPPA* | IIS |
| rs3789280 | A/T | 9 | 117993193 | *PAPPA* | IIS |
| rs398400 | A/C | 9 | 118028307 | *PAPPA* | IIS |
| rs449807 | A/T | 9 | 118005570 | *PAPPA* | IIS |
| rs4837525 | A/G | 9 | 118078379 | *PAPPA* | IIS |
| rs7020782 | A/C | 9 | 118146702 | *PAPPA* | IIS |
| rs7025886 | A/C | 9 | 117982260 | *PAPPA* | IIS |
| rs731146 | A/C | 9 | 118134366 | *PAPPA* | IIS |
| rs7469968 | A/T | 9 | 118019870 | *PAPPA* | IIS |
| rs7869550 | A/G | 9 | 118174617 | *PAPPA* | IIS |
| rs978201 | A/T | 9 | 118196851 | *PAPPA* | IIS |
| rs161802 | A/C | 1 | 7965413 | *PARK7* | PRO/ANTIOXI |
| rs178932 | A/G | 1 | 7952887 | *PARK7* | PRO/ANTIOXI |
| rs225092 | C/G | 1 | 7958662 | *PARK7* | PRO/ANTIOXI |
| rs225119 | A/G | 1 | 7966948 | *PARK7* | PRO/ANTIOXI |
| rs226242 | A/G | 1 | 7956055 | *PARK7* | PRO/ANTIOXI |
| rs226258 | A/C | 1 | 7963939 | *PARK7* | PRO/ANTIOXI |
| rs4908488 | A/G | 1 | 7947179 | *PARK7* | PRO/ANTIOXI |
| rs1136410 | A/G | 1 | 224621925 | *PARP1* | DNA REPAIR |
| rs1805410 | A/G | 1 | 224635288 | *PARP1* | DNA REPAIR |
| rs1805414 | A/G | 1 | 224639987 | *PARP1* | DNA REPAIR |
| rs2271347 | G/A | 1 | 224616121 | *PARP1* | DNA REPAIR |
| rs3219110 | A/G | 1 | 224624501 | *PARP1* | DNA REPAIR |
| rs3219142 | A/G | 1 | 224618691 | *PARP1* | DNA REPAIR |
| rs7542788 | A/G | 1 | 224651926 | *PARP1* | DNA REPAIR |
| rs17349 | A/G | 20 | 5047516 | *PCNA* | DNA REPAIR |
| rs25406 | G/A | 20 | 5047636 | *PCNA* | DNA REPAIR |
| rs3729558 | C/G | 20 | 5043321 | *PCNA* | DNA REPAIR |
| rs8125170 | A/G | 20 | 5054287 | *PCNA* | DNA REPAIR |
| rs1005273 | A/G | 16 | 2585966 | *PDPK1* | IIS |
| rs10513055 | A/C | 3 | 139934042 | *PI3KCB* | IIS |
| rs361072 | A/G | 3 | 139961234 | *PI3KCB* | IIS |
| rs11631663 | A/G | 15 | 62904700 | *PIF1* | DNA REPAIR |
| rs17802279 | A/T | 15 | 62895557 | *PIF1* | DNA REPAIR |
| rs3743046 | A/C | 15 | 62904875 | *PIF1* | DNA REPAIR |
| rs1233255 | A/C | 2 | 190414337 | *PMS1* | DNA REPAIR |
| rs1233258 | A/G | 2 | 190415279 | *PMS1* | DNA REPAIR |
| rs1233284 | A/G | 2 | 190398016 | *PMS1* | DNA REPAIR |
| rs17806132 | G/A | 2 | 190416532 | *PMS1* | DNA REPAIR |
| rs256552 | A/G | 2 | 190450619 | *PMS1* | DNA REPAIR |
| rs5743100 | C/A | 2 | 190414098 | *PMS1* | DNA REPAIR |
| rs11769380 | A/G | 7 | 6009048 | *PMS2* | DNA REPAIR |
| rs12112229 | A/C | 7 | 6003041 | *PMS2* | DNA REPAIR |
| rs2286680 | G/A | 7 | 6002033 | *PMS2* | DNA REPAIR |
| rs2345060 | A/G | 7 | 6005655 | *PMS2* | DNA REPAIR |
| rs7811924 | G/A | 7 | 5991662 | *PMS2* | DNA REPAIR |
| rs2953983 | A/G | 8 | 42332413 | *POLB* | DNA REPAIR |
| rs1673041 | C/A | 19 | 55601201 | *POLD1* | DNA REPAIR |
| rs2546551 | A/G | 19 | 55588172 | *POLD1* | DNA REPAIR |
| rs3219281 | G/A | 19 | 55578899 | *POLD1* | DNA REPAIR |
| rs3219337 | A/G | 19 | 55589419 | *POLD1* | DNA REPAIR |
| rs4883539 | A/G | 12 | 131723523 | *POLE* | DNA REPAIR |
| rs4883617 | A/G | 12 | 131726690 | *POLE* | DNA REPAIR |
| rs5744873 | A/C | 12 | 131745405 | *POLE* | DNA REPAIR |
| rs5744897 | G/A | 12 | 131736904 | *POLE* | DNA REPAIR |
| rs5744934 | A/G | 12 | 131730599 | *POLE* | DNA REPAIR |
| rs5744941 | T/A | 12 | 131730062 | *POLE* | DNA REPAIR |
| rs5744990 | G/A | 12 | 131722655 | *POLE* | DNA REPAIR |
| rs2072266 | A/G | 15 | 87667958 | *POLG* | DNA REPAIR |
| rs2351000 | A/G | 15 | 87670737 | *POLG* | DNA REPAIR |
| rs3087374 | A/C | 15 | 87660998 | *POLG* | DNA REPAIR |
| rs3176205 | A/G | 15 | 87666696 | *POLG* | DNA REPAIR |
| rs3176208 | A/C | 15 | 87665767 | *POLG* | DNA REPAIR |
| rs2283575 | G/A | 19 | 580303 | *POLRMT* | DNA REPAIR |
| rs2049649 | A/G | 7 | 94787265 | *PON1* | PRO/ANTIOXI |
| rs2074351 | A/G | 7 | 94785735 | *PON1* | PRO/ANTIOXI |
| rs2237583 | A/G | 7 | 94788113 | *PON1* | PRO/ANTIOXI |
| rs2237584 | A/G | 7 | 94788773 | *PON1* | PRO/ANTIOXI |
| rs2299257 | A/C | 7 | 94780701 | *PON1* | PRO/ANTIOXI |
| rs2299261 | A/G | 7 | 94787599 | *PON1* | PRO/ANTIOXI |
| rs2299262 | A/G | 7 | 94787864 | *PON1* | PRO/ANTIOXI |
| rs2374983 | A/G | 7 | 94709660 | *PON1* | PRO/ANTIOXI |
| rs3735590 | A/G | 7 | 94765431 | *PON1* | PRO/ANTIOXI |
| rs3917521 | A/G | 7 | 94779351 | *PON1* | PRO/ANTIOXI |
| rs3917550 | A/G | 7 | 94772509 | *PON1* | PRO/ANTIOXI |
| rs3917556 | A/G | 7 | 94771125 | *PON1* | PRO/ANTIOXI |
| rs662 | A/G | 7 | 94775382 | *PON1* | PRO/ANTIOXI |
| rs757158 | A/G | 7 | 94793464 | *PON1* | PRO/ANTIOXI |
| rs854551 | A/G | 7 | 94765613 | *PON1* | PRO/ANTIOXI |
| rs854552 | A/G | 7 | 94765860 | *PON1* | PRO/ANTIOXI |
| rs854555 | A/C | 7 | 94768327 | *PON1* | PRO/ANTIOXI |
| rs854565 | A/G | 7 | 94786280 | *PON1* | PRO/ANTIOXI |
| rs854567 | A/G | 7 | 94786720 | *PON1* | PRO/ANTIOXI |
| rs854568 | A/G | 7 | 94787737 | *PON1* | PRO/ANTIOXI |
| rs854570 | A/C | 7 | 94790628 | *PON1* | PRO/ANTIOXI |
| rs854573 | A/G | 7 | 94792799 | *PON1* | PRO/ANTIOXI |
| rs10487133 | A/C | 7 | 94873886 | *PON2* | PRO/ANTIOXI |
| rs12026 | C/G | 7 | 94878952 | *PON2* | PRO/ANTIOXI |
| rs12669182 | A/G | 7 | 94881088 | *PON2* | PRO/ANTIOXI |
| rs17879277 | A/C | 7 | 94868568 | *PON2* | PRO/ANTIOXI |
| rs2299267 | A/G | 7 | 94899857 | *PON2* | PRO/ANTIOXI |
| rs2375005 | A/T | 7 | 94874812 | *PON2* | PRO/ANTIOXI |
| rs43037 | A/G | 7 | 94904942 | *PON2* | PRO/ANTIOXI |
| rs4729189 | A/T | 7 | 94895895 | *PON2* | PRO/ANTIOXI |
| rs730365 | A/G | 7 | 94900936 | *PON2* | PRO/ANTIOXI |
| rs7493 | C/G | 7 | 94872711 | *PON2* | PRO/ANTIOXI |
| rs7802018 | A/G | 7 | 94898249 | *PON2* | PRO/ANTIOXI |
| rs7803148 | A/G | 7 | 94885766 | *PON2* | PRO/ANTIOXI |
| rs10487132 | A/G | 7 | 94858241 | *PON3* | PRO/ANTIOXI |
| rs11767787 | A/G | 7 | 94864689 | *PON3* | PRO/ANTIOXI |
| rs2057682 | C/G | 7 | 94828700 | *PON3* | PRO/ANTIOXI |
| rs2072200 | C/G | 7 | 94864096 | *PON3* | PRO/ANTIOXI |
| rs10228682 | A/G | 7 | 124325272 | *POT1* | DNA REPAIR |
| rs10271646 | A/G | 7 | 124336809 | *POT1* | DNA REPAIR |
| rs11768553 | A/G | 7 | 124313384 | *POT1* | DNA REPAIR |
| rs11972248 | A/G | 7 | 124276246 | *POT1* | DNA REPAIR |
| rs929365 | A/G | 7 | 124251667 | *POT1* | DNA REPAIR |
| rs10511134 | A/T | 3 | 87408911 | *POU1F1* | IIS |
| rs12486159 | A/T | 3 | 87394583 | *POU1F1* | IIS |
| rs177292 | A/G | 3 | 87413028 | *POU1F1* | IIS |
| rs2633674 | G/A | 3 | 87411055 | *POU1F1* | IIS |
| rs300982 | G/A | 3 | 87408443 | *POU1F1* | IIS |
| rs300994 | G/A | 3 | 87411438 | *POU1F1* | IIS |
| rs9824592 | A/C | 3 | 87398490 | *POU1F1* | IIS |
| rs11198811 | C/G | 10 | 120927273 | *PRDX3* | PRO/ANTIOXI |
| rs1553850 | A/T | 10 | 120928814 | *PRDX3* | PRO/ANTIOXI |
| rs3377 | A/C | 10 | 120917534 | *PRDX3* | PRO/ANTIOXI |
| rs3740562 | A/G | 10 | 120926691 | *PRDX3* | PRO/ANTIOXI |
| rs4752257 | A/C | 10 | 120923551 | *PRDX3* | PRO/ANTIOXI |
| rs7768 | C/G | 10 | 120917783 | *PRDX3* | PRO/ANTIOXI |
| rs10109984 | A/G | 8 | 48966228 | *PRKDC* | DNA REPAIR |
| rs2213178 | A/G | 8 | 48979269 | *PRKDC* | DNA REPAIR |
| rs4278157 | G/A | 8 | 48901858 | *PRKDC* | DNA REPAIR |
| rs4521758 | G/A | 8 | 48904123 | *PRKDC* | DNA REPAIR |
| rs4873772 | A/G | 8 | 49021486 | *PRKDC* | DNA REPAIR |
| rs8178017 | G/A | 8 | 49014778 | *PRKDC* | DNA REPAIR |
| rs8178068 | G/A | 8 | 48978431 | *PRKDC* | DNA REPAIR |
| rs8178179 | A/C | 8 | 48907680 | *PRKDC* | DNA REPAIR |
| rs4431364 | G/C | 5 | 177354664 | *PROP1* | IIS |
| rs4604209 | A/G | 5 | 177351962 | *PROP1* | IIS |
| rs4610479 | C/G | 5 | 177355142 | *PROP1* | IIS |
| rs6883364 | A/G | 5 | 177351360 | *PROP1* | IIS |
| rs6890425 | A/G | 5 | 177359136 | *PROP1* | IIS |
| rs11202596 | G/A | 10 | 89637134 | *PTEN* | IIS |
| rs11202600 | G/C | 10 | 89672813 | *PTEN* | IIS |
| rs1234220 | A/G | 10 | 89635453 | *PTEN* | IIS |
| rs17431184 | A/G | 10 | 89710231 | *PTEN* | IIS |
| rs1903858 | A/G | 10 | 89643666 | *PTEN* | IIS |
| rs1903860 | A/G | 10 | 89610190 | *PTEN* | IIS |
| rs2736627 | A/G | 10 | 89711074 | *PTEN* | IIS |
| rs13045716 | C/G | 20 | 48595648 | *PTPN1* | IIS |
| rs2038526 | A/G | 20 | 48619056 | *PTPN1* | IIS |
| rs2426164 | A/G | 20 | 48631734 | *PTPN1* | IIS |
| rs6063534 | A/G | 20 | 48622575 | *PTPN1* | IIS |
| rs6067484 | A/G | 20 | 48586190 | *PTPN1* | IIS |
| rs6126033 | G/A | 20 | 48579516 | *PTPN1* | IIS |
| rs6512652 | G/A | 20 | 48595515 | *PTPN1* | IIS |
| rs10119641 | A/G | 9 | 109082068 | *RAD23B* | DNA REPAIR |
| rs10739239 | A/T | 9 | 109101964 | *RAD23B* | DNA REPAIR |
| rs10816492 | A/G | 9 | 109123748 | *RAD23B* | DNA REPAIR |
| rs11573631 | A/G | 9 | 109095700 | *RAD23B* | DNA REPAIR |
| rs11573709 | A/G | 9 | 109125076 | *RAD23B* | DNA REPAIR |
| rs11573711 | A/G | 9 | 109125389 | *RAD23B* | DNA REPAIR |
| rs1323808 | A/G | 9 | 109124525 | *RAD23B* | DNA REPAIR |
| rs13299620 | A/C | 9 | 109093647 | *RAD23B* | DNA REPAIR |
| rs1805329 | A/G | 9 | 109124149 | *RAD23B* | DNA REPAIR |
| rs4978820 | G/A | 9 | 109097683 | *RAD23B* | DNA REPAIR |
| rs7023656 | A/G | 9 | 109115258 | *RAD23B* | DNA REPAIR |
| rs7030169 | A/C | 9 | 109093889 | *RAD23B* | DNA REPAIR |
| rs17772583 | A/G | 5 | 131981409 | *RAD50* | DNA REPAIR |
| rs2237060 | A/C | 5 | 131998784 | *RAD50* | DNA REPAIR |
| rs2706370 | A/C | 5 | 131960915 | *RAD50* | DNA REPAIR |
| rs11633269 | A/G | 15 | 38807159 | *RAD51* | DNA REPAIR |
| rs1801320 | G/C | 15 | 38774820 | *RAD51* | DNA REPAIR |
| rs2619681 | A/G | 15 | 38776313 | *RAD51* | DNA REPAIR |
| rs5030783 | A/G | 15 | 38771681 | *RAD51* | DNA REPAIR |
| rs10744729 | A/C | 12 | 894855 | *RAD52* | DNA REPAIR |
| rs11064598 | A/T | 12 | 917727 | *RAD52* | DNA REPAIR |
| rs11064613 | A/G | 12 | 933419 | *RAD52* | DNA REPAIR |
| rs12822733 | C/G | 12 | 926291 | *RAD52* | DNA REPAIR |
| rs12827646 | A/G | 12 | 898576 | *RAD52* | DNA REPAIR |
| rs7311151 | A/G | 12 | 920515 | *RAD52* | DNA REPAIR |
| rs7311263 | A/C | 12 | 922654 | *RAD52* | DNA REPAIR |
| rs7962050 | A/G | 12 | 901715 | *RAD52* | DNA REPAIR |
| rs10789488 | A/G | 1 | 46491492 | *RAD54L* | DNA REPAIR |
| rs12146051 | G/C | 1 | 46515539 | *RAD54L* | DNA REPAIR |
| rs12410307 | A/G | 1 | 46502985 | *RAD54L* | DNA REPAIR |
| rs17102086 | A/G | 1 | 46495526 | *RAD54L* | DNA REPAIR |
| rs17102087 | A/G | 1 | 46499891 | *RAD54L* | DNA REPAIR |
| rs2295465 | A/C | 1 | 46498644 | *RAD54L* | DNA REPAIR |
| rs9793263 | A/G | 1 | 46494976 | *RAD54L* | DNA REPAIR |
| rs1061627 | A/G | 12 | 21545674 | *RecQL1* | DNA REPAIR |
| rs10841833 | A/C | 12 | 21528492 | *RecQL1* | DNA REPAIR |
| rs12231436 | A/C | 12 | 21540806 | *RecQL1* | DNA REPAIR |
| rs1860947 | A/C | 12 | 21545911 | *RecQL1* | DNA REPAIR |
| rs2110159 | A/G | 12 | 21540427 | *RecQL1* | DNA REPAIR |
| rs2192170 | G/A | 12 | 21524303 | *RecQL1* | DNA REPAIR |
| rs2284392 | A/G | 12 | 21523201 | *RecQL1* | DNA REPAIR |
| rs2284393 | A/G | 12 | 21538604 | *RecQL1* | DNA REPAIR |
| rs2300211 | A/C | 12 | 21544386 | *RecQL1* | DNA REPAIR |
| rs3213212 | A/G | 12 | 21526499 | *RecQL1* | DNA REPAIR |
| rs6487233 | A/G | 12 | 21529837 | *RecQL1* | DNA REPAIR |
| rs7307519 | C/G | 12 | 21525764 | *RecQL1* | DNA REPAIR |
| rs917855 | A/G | 12 | 21521413 | *RecQL1* | DNA REPAIR |
| rs4251689 | A/G | 8 | 145711938 | *RECQL4* | DNA REPAIR |
| rs820157 | A/G | 17 | 71173006 | *RecQL5* | DNA REPAIR |
| rs12644680 | A/G | 4 | 39047276 | *RFC1* | DNA REPAIR |
| rs13147094 | A/G | 4 | 38987277 | *RFC1* | DNA REPAIR |
| rs16995255 | C/G | 4 | 39041083 | *RFC1* | DNA REPAIR |
| rs17584703 | A/G | 4 | 39041251 | *RFC1* | DNA REPAIR |
| rs2066789 | A/G | 4 | 38984582 | *RFC1* | DNA REPAIR |
| rs2306596 | A/C | 4 | 39020335 | *RFC1* | DNA REPAIR |
| rs2306597 | G/A | 4 | 38973595 | *RFC1* | DNA REPAIR |
| rs3733282 | A/G | 4 | 38965339 | *RFC1* | DNA REPAIR |
| rs6829064 | A/G | 4 | 38968207 | *RFC1* | DNA REPAIR |
| rs11656253 | A/G | 17 | 1708196 | *RPA1* | DNA REPAIR |
| rs11867830 | A/G | 17 | 1707129 | *RPA1* | DNA REPAIR |
| rs16951710 | A/G | 17 | 1704953 | *RPA1* | DNA REPAIR |
| rs17292175 | A/G | 17 | 1731525 | *RPA1* | DNA REPAIR |
| rs17339284 | A/G | 17 | 1746408 | *RPA1* | DNA REPAIR |
| rs17734 | G/A | 17 | 1747894 | *RPA1* | DNA REPAIR |
| rs2270412 | G/A | 17 | 1738924 | *RPA1* | DNA REPAIR |
| rs2287320 | A/G | 17 | 1703387 | *RPA1* | DNA REPAIR |
| rs4790830 | A/T | 17 | 1701130 | *RPA1* | DNA REPAIR |
| rs4790838 | A/G | 17 | 1746837 | *RPA1* | DNA REPAIR |
| rs5030740 | A/G | 17 | 1747350 | *RPA1* | DNA REPAIR |
| rs5030755 | A/G | 17 | 1729702 | *RPA1* | DNA REPAIR |
| rs8067195 | A/G | 17 | 1725022 | *RPA1* | DNA REPAIR |
| rs9909163 | G/A | 17 | 1735396 | *RPA1* | DNA REPAIR |
| rs2070424 | A/G | 21 | 31961191 | *SOD1* | PRO/ANTIOXI |
| rs4998557 | A/G | 21 | 31956763 | *SOD1* | PRO/ANTIOXI |
| rs2842980 | A/T | 6 | 160020106 | *SOD2* | PRO/ANTIOXI |
| rs5746136 | A/G | 6 | 160023074 | *SOD2* | PRO/ANTIOXI |
| rs5746151 | A/G | 6 | 160021310 | *SOD2* | PRO/ANTIOXI |
| rs911847 | A/G | 6 | 159988958 | *SOD2* | PRO/ANTIOXI |
| rs17878863 | A/G | 4 | 24406916 | *SOD3* | PRO/ANTIOXI |
| rs2284659 | A/C | 4 | 24403895 | *SOD3* | PRO/ANTIOXI |
| rs2855262 | A/G | 4 | 24411074 | *SOD3* | PRO/ANTIOXI |
| rs699473 | A/G | 4 | 24405901 | *SOD3* | PRO/ANTIOXI |
| rs800442 | C/G | 4 | 24402642 | *SOD3* | PRO/ANTIOXI |
| rs8192287 | A/C | 4 | 24405666 | *SOD3* | PRO/ANTIOXI |
| rs2008022 | A/C | 20 | 579425 | *SRXN1* | PRO/ANTIOXI |
| rs6053666 | A/G | 20 | 576890 | *SRXN1* | PRO/ANTIOXI |
| rs6085283 | A/G | 20 | 580959 | *SRXN1* | PRO/ANTIOXI |
| rs7269823 | A/G | 20 | 579117 | *SRXN1* | PRO/ANTIOXI |
| rs10513819 | G/A | 3 | 188874192 | *SST* | IIS |
| rs17796004 | A/G | 3 | 188868748 | *SST* | IIS |
| rs2162189 | A/G | 3 | 188873755 | *SST* | IIS |
| rs7624906 | A/G | 3 | 188872330 | *SST* | IIS |
| rs1466113 | C/G | 17 | 68676913 | *SSTR2* | IIS |
| rs7210080 | A/G | 17 | 68678697 | *SSTR2* | IIS |
| rs7224362 | A/G | 17 | 68679136 | *SSTR2* | IIS |
| rs728291 | A/C | 17 | 68675383 | *SSTR2* | IIS |
| rs2063047 | A/G | 10 | 70617764 | *SUPV3L1* | DNA REPAIR |
| rs4746821 | A/C | 10 | 70638205 | *SUPV3L1* | DNA REPAIR |
| rs6480392 | G/A | 10 | 70618688 | *SUPV3L1* | DNA REPAIR |
| rs7893492 | G/A | 10 | 70630454 | *SUPV3L1* | DNA REPAIR |
| rs10095169 | A/G | 8 | 74092275 | *TERF1* | DNA REPAIR |
| rs10098931 | A/C | 8 | 74079430 | *TERF1* | DNA REPAIR |
| rs2929586 | A/G | 8 | 74087966 | *TERF1* | DNA REPAIR |
| rs2975842 | A/G | 8 | 74088145 | *TERF1* | DNA REPAIR |
| rs6982126 | A/G | 8 | 74102177 | *TERF1* | DNA REPAIR |
| rs251796 | A/G | 16 | 67952935 | *TERF2* | DNA REPAIR |
| rs34295116 | G/A | 16 | 67952713 | *TERF2* | DNA REPAIR |
| rs35439397 | A/G | 16 | 67959753 | *TERF2* | DNA REPAIR |
| rs8061352 | A/T | 16 | 67976532 | *TERF2* | DNA REPAIR |
| rs9925619 | C/G | 16 | 67949215 | *TERF2* | DNA REPAIR |
| rs1865493 | C/G | 16 | 74239244 | *TERF2IP* | DNA REPAIR |
| rs10069690 | A/G | 5 | 1332790 | *TERT* | DNA REPAIR |
| rs13167280 | A/G | 5 | 1333477 | *TERT* | DNA REPAIR |
| rs2735940 | A/G | 5 | 1349486 | *TERT* | DNA REPAIR |
| rs2853668 | A/C | 5 | 1353025 | *TERT* | DNA REPAIR |
| rs33954691 | G/A | 5 | 1308520 | *TERT* | DNA REPAIR |
| rs3891054 | C/G | 5 | 1320202 | *TERT* | DNA REPAIR |
| rs4246742 | A/T | 5 | 1320356 | *TERT* | DNA REPAIR |
| rs10826176 | A/G | 10 | 59811543 | *TFAM* | DNA REPAIR |
| rs11006127 | A/C | 10 | 59813027 | *TFAM* | DNA REPAIR |
| rs11006132 | A/G | 10 | 59826172 | *TFAM* | DNA REPAIR |
| rs12355138 | A/G | 10 | 59810715 | *TFAM* | DNA REPAIR |
| rs16912200 | A/G | 10 | 59826601 | *TFAM* | DNA REPAIR |
| rs2279339 | C/A | 10 | 59815004 | *TFAM* | DNA REPAIR |
| rs3876 | G/A | 10 | 59825797 | *TFAM* | DNA REPAIR |
| rs12951053 | A/C | 17 | 7518132 | *TP53* | DNA REPAIR |
| rs2078486 | A/G | 17 | 7523808 | *TP53* | DNA REPAIR |
| rs8079544 | G/A | 17 | 7520777 | *TP53* | DNA REPAIR |
| rs12159295 | A/G | 22 | 35195590 | *TXN2* | PRO/ANTIOXI |
| rs5756202 | A/C | 22 | 35200791 | *TXN2* | PRO/ANTIOXI |
| rs8139906 | C/G | 22 | 35198206 | *TXN2* | PRO/ANTIOXI |
| rs9619607 | A/G | 22 | 35200565 | *TXN2* | PRO/ANTIOXI |
| rs10047589 | A/G | 12 | 103268518 | *TXNRD1* | PRO/ANTIOXI |
| rs10778318 | A/G | 12 | 103195636 | *TXNRD1* | PRO/ANTIOXI |
| rs10861169 | A/G | 12 | 103134770 | *TXNRD1* | PRO/ANTIOXI |
| rs10861171 | A/G | 12 | 103141908 | *TXNRD1* | PRO/ANTIOXI |
| rs10861197 | A/G | 12 | 103230161 | *TXNRD1* | PRO/ANTIOXI |
| rs10861203 | A/G | 12 | 103260828 | *TXNRD1* | PRO/ANTIOXI |
| rs17202060 | A/G | 12 | 103254976 | *TXNRD1* | PRO/ANTIOXI |
| rs4445711 | A/G | 12 | 103160731 | *TXNRD1* | PRO/ANTIOXI |
| rs4964728 | A/G | 12 | 103173863 | *TXNRD1* | PRO/ANTIOXI |
| rs4964735 | A/G | 12 | 103180256 | *TXNRD1* | PRO/ANTIOXI |
| rs4964778 | C/G | 12 | 103210194 | *TXNRD1* | PRO/ANTIOXI |
| rs7301631 | A/G | 12 | 103197392 | *TXNRD1* | PRO/ANTIOXI |
| rs7310505 | A/C | 12 | 103178678 | *TXNRD1* | PRO/ANTIOXI |
| rs7310815 | C/G | 12 | 103137978 | *TXNRD1* | PRO/ANTIOXI |
| rs7962759 | C/G | 12 | 103263146 | *TXNRD1* | PRO/ANTIOXI |
| rs12502572 | A/G | 4 | 141704584 | *UCP1* | PRO/ANTIOXI |
| rs1472268 | A/T | 4 | 141712393 | *UCP1* | PRO/ANTIOXI |
| rs1800592 | A/G | 4 | 141713411 | *UCP1* | PRO/ANTIOXI |
| rs2071416 | A/C | 4 | 141708182 | *UCP1* | PRO/ANTIOXI |
| rs3811790 | A/C | 4 | 141711026 | *UCP1* | PRO/ANTIOXI |
| rs6536991 | A/G | 4 | 141701031 | *UCP1* | PRO/ANTIOXI |
| rs6822807 | A/G | 4 | 141707198 | *UCP1* | PRO/ANTIOXI |
| rs7687015 | A/C | 4 | 141713327 | *UCP1* | PRO/ANTIOXI |
| rs7688743 | A/G | 4 | 141708245 | *UCP1* | PRO/ANTIOXI |
| rs591758 | C/G | 11 | 73375708 | *UCP2* | PRO/ANTIOXI |
| rs659366 | A/G | 11 | 73372402 | *UCP2* | PRO/ANTIOXI |
| rs7109266 | A/G | 11 | 73375186 | *UCP2* | PRO/ANTIOXI |
| rs11235972 | A/G | 11 | 73394722 | *UCP3* | PRO/ANTIOXI |
| rs1685354 | A/G | 11 | 73391239 | *UCP3* | PRO/ANTIOXI |
| rs3781907 | A/G | 11 | 73394117 | *UCP3* | PRO/ANTIOXI |
| rs647126 | A/G | 11 | 73389668 | *UCP3* | PRO/ANTIOXI |
| rs246079 | A/G | 12 | 108031443 | *UNG* | DNA REPAIR |
| rs2569987 | A/G | 12 | 108025554 | *UNG* | DNA REPAIR |
| rs759561 | A/G | 12 | 108017802 | *UNG* | DNA REPAIR |
| rs3760841 | A/G | 19 | 34397623 | *UQCRFS1* | PRO/ANTIOXI |
| rs11574214 | T/A | 8 | 31052816 | *WRN* | DNA REPAIR |
| rs11574218 | A/C | 8 | 31057332 | *WRN* | DNA REPAIR |
| rs11574304 | G/A | 8 | 31093353 | *WRN* | DNA REPAIR |
| rs11574309 | A/G | 8 | 31094542 | *WRN* | DNA REPAIR |
| rs13251813 | G/A | 8 | 31083753 | *WRN* | DNA REPAIR |
| rs13269094 | A/C | 8 | 31015693 | *WRN* | DNA REPAIR |
| rs1346044 | A/G | 8 | 31144196 | *WRN* | DNA REPAIR |
| rs1800391 | G/A | 8 | 31058246 | *WRN* | DNA REPAIR |
| rs1882928 | A/G | 8 | 31143364 | *WRN* | DNA REPAIR |
| rs2230009 | G/A | 8 | 31041477 | *WRN* | DNA REPAIR |
| rs2725344 | A/G | 8 | 31037640 | *WRN* | DNA REPAIR |
| rs2725351 | A/G | 8 | 31096988 | *WRN* | DNA REPAIR |
| rs6982140 | A/G | 8 | 31118970 | *WRN* | DNA REPAIR |
| rs6982948 | A/C | 8 | 31149817 | *WRN* | DNA REPAIR |
| rs1042039 | A/G | 2 | 31411810 | *XDH* | PRO/ANTIOXI |
| rs1366811 | A/G | 2 | 31423573 | *XDH* | PRO/ANTIOXI |
| rs1429372 | A/G | 2 | 31479889 | *XDH* | PRO/ANTIOXI |
| rs169596 | A/G | 2 | 31419628 | *XDH* | PRO/ANTIOXI |
| rs17038412 | A/T | 2 | 31438719 | *XDH* | PRO/ANTIOXI |
| rs2043013 | A/C | 2 | 31445827 | *XDH* | PRO/ANTIOXI |
| rs206812 | A/G | 2 | 31491373 | *XDH* | PRO/ANTIOXI |
| rs206851 | A/G | 2 | 31467586 | *XDH* | PRO/ANTIOXI |
| rs206860 | A/G | 2 | 31476308 | *XDH* | PRO/ANTIOXI |
| rs2073316 | A/G | 2 | 31464533 | *XDH* | PRO/ANTIOXI |
| rs207444 | A/G | 2 | 31417301 | *XDH* | PRO/ANTIOXI |
| rs207455 | A/G | 2 | 31421433 | *XDH* | PRO/ANTIOXI |
| rs2163059 | A/G | 2 | 31456638 | *XDH* | PRO/ANTIOXI |
| rs2281547 | A/G | 2 | 31452327 | *XDH* | PRO/ANTIOXI |
| rs494852 | A/G | 2 | 31478340 | *XDH* | PRO/ANTIOXI |
| rs6718606 | C/G | 2 | 31457111 | *XDH* | PRO/ANTIOXI |
| rs7574920 | C/G | 2 | 31439153 | *XDH* | PRO/ANTIOXI |
| rs994727 | A/C | 2 | 31441015 | *XDH* | PRO/ANTIOXI |
| rs2805835 | G/C | 9 | 99484772 | *XPA* | DNA REPAIR |
| rs2808667 | A/G | 9 | 99482627 | *XPA* | DNA REPAIR |
| rs2808668 | A/G | 9 | 99492256 | *XPA* | DNA REPAIR |
| rs3176658 | G/A | 9 | 99493684 | *XPA* | DNA REPAIR |
| rs3176683 | A/G | 9 | 99488438 | *XPA* | DNA REPAIR |
| rs3176689 | A/T | 9 | 99487617 | *XPA* | DNA REPAIR |
| rs3176748 | A/G | 9 | 99478165 | *XPA* | DNA REPAIR |
| rs3176757 | A/G | 9 | 99476879 | *XPA* | DNA REPAIR |
| rs13099160 | A/G | 3 | 14177803 | *XPC* | DNA REPAIR |
| rs2228000 | A/G | 3 | 14174889 | *XPC* | DNA REPAIR |
| rs2733534 | C/G | 3 | 14163763 | *XPC* | DNA REPAIR |
| rs2733537 | A/G | 3 | 14186105 | *XPC* | DNA REPAIR |
| rs3731108 | A/G | 3 | 14182810 | *XPC* | DNA REPAIR |
| rs3731143 | A/G | 3 | 14172547 | *XPC* | DNA REPAIR |
| rs9653966 | A/C | 3 | 14169431 | *XPC* | DNA REPAIR |
| rs1799782 | G/A | 19 | 48749414 | *XRCC1* | DNA REPAIR |
| rs2023614 | C/G | 19 | 48756705 | *XRCC1* | DNA REPAIR |
| rs2682585 | G/A | 19 | 48773128 | *XRCC1* | DNA REPAIR |
| rs2682587 | A/C | 19 | 48774269 | *XRCC1* | DNA REPAIR |
| rs2854501 | A/G | 19 | 48751841 | *XRCC1* | DNA REPAIR |
| rs2854508 | A/T | 19 | 48768004 | *XRCC1* | DNA REPAIR |
| rs2854510 | A/G | 19 | 48766052 | *XRCC1* | DNA REPAIR |
| rs3213266 | A/G | 19 | 48767476 | *XRCC1* | DNA REPAIR |
| rs3213403 | A/G | 19 | 48738568 | *XRCC1* | DNA REPAIR |
| rs13006837 | A/G | 2 | 216767499 | *XRCC5* | DNA REPAIR |
| rs1364726 | A/G | 2 | 216731058 | *XRCC5* | DNA REPAIR |
| rs16855458 | C/A | 2 | 216719066 | *XRCC5* | DNA REPAIR |
| rs16855489 | A/G | 2 | 216726501 | *XRCC5* | DNA REPAIR |
| rs207905 | A/C | 2 | 216719857 | *XRCC5* | DNA REPAIR |
| rs207910 | A/G | 2 | 216726667 | *XRCC5* | DNA REPAIR |
| rs207924 | A/G | 2 | 216741539 | *XRCC5* | DNA REPAIR |
| rs207929 | A/G | 2 | 216744917 | *XRCC5* | DNA REPAIR |
| rs207943 | C/G | 2 | 216752630 | *XRCC5* | DNA REPAIR |
| rs2303400 | A/G | 2 | 216711397 | *XRCC5* | DNA REPAIR |
| rs3770500 | A/T | 2 | 216754564 | *XRCC5* | DNA REPAIR |
| rs3821107 | A/G | 2 | 216739573 | *XRCC5* | DNA REPAIR |
| rs3834 | G/A | 2 | 216774948 | *XRCC5* | DNA REPAIR |
| rs4674066 | A/G | 2 | 216768209 | *XRCC5* | DNA REPAIR |
| rs668844 | A/C | 2 | 216729847 | *XRCC5* | DNA REPAIR |
| rs705649 | A/G | 2 | 216689431 | *XRCC5* | DNA REPAIR |
| rs828704 | A/C | 2 | 216701856 | *XRCC5* | DNA REPAIR |
| rs828910 | A/G | 2 | 216685273 | *XRCC5* | DNA REPAIR |
| rs1011980 | A/G | 5 | 82429305 | *XRRC4* | DNA REPAIR |
| rs10474079 | A/G | 5 | 82409673 | *XRRC4* | DNA REPAIR |
| rs1056503 | A/C | 5 | 82684733 | *XRRC4* | DNA REPAIR |
| rs1193695 | A/G | 5 | 82578842 | *XRRC4* | DNA REPAIR |
| rs13178127 | A/G | 5 | 82625188 | *XRRC4* | DNA REPAIR |
| rs177297 | G/A | 5 | 82589894 | *XRRC4* | DNA REPAIR |
| rs2075685 | A/C | 5 | 82408421 | *XRRC4* | DNA REPAIR |
| rs2731852 | A/G | 5 | 82544166 | *XRRC4* | DNA REPAIR |
| rs301275 | T/A | 5 | 82598817 | *XRRC4* | DNA REPAIR |
| rs301287 | G/A | 5 | 82640966 | *XRRC4* | DNA REPAIR |
| rs6452524 | A/G | 5 | 82469537 | *XRRC4* | DNA REPAIR |
| rs7711825 | C/A | 5 | 82557374 | *XRRC4* | DNA REPAIR |
| rs7720588 | A/G | 5 | 82412682 | *XRRC4* | DNA REPAIR |
| rs10493112 | A/C | 1 | 42931017 | *YBX1* | DNA REPAIR |
| rs10493113 | A/G | 1 | 42931097 | *YBX1* | DNA REPAIR |
| rs11210696 | A/C | 1 | 42927649 | *YBX1* | DNA REPAIR |
| rs11210698 | A/G | 1 | 42941594 | *YBX1* | DNA REPAIR |
| rs12030724 | A/T | 1 | 42932605 | *YBX1* | DNA REPAIR |
| rs3895305 | A/C | 1 | 42927079 | *YBX1* | DNA REPAIR |
| rs6659400 | A/G | 1 | 42918795 | *YBX1* | DNA REPAIR |

Table 3S: List of all analysed SNPs, classified by gene and respect to the three main pathway DNA repair, Insulin-Igf Signaling (IIS) and Pro/Antioxidant Response.
