# Supplementary material for: The genetic component of human longevity: New insights from the analysis of pathway‐based SNP‐SNP interactions
Source: Aging Cell. 2018 Mar 25;17(3):e12755. doi: 10.1111/acel.12755 (PMC5946073; doi:10.1111/acel.12755)
Supplement: Supplementary file 4 [file ACEL-17-e12755-s004.docx]

| **SNP** | **VARIANT** | **CHR** | **POSITION** | **GENE** | **PATHWAY** | **dbSNP FUNCTIONAL ANNOTATION** | **FUNCTIONAL IMPACT OF THE SNP** |  |  |  |  |  |  |
| --- | --- | --- | --- | --- | --- | --- | --- | --- | --- | --- | --- | --- | --- |
| rs2465661 | A/G | 2 | 201208876 | AOX1 | PRO/ANTIOXI | Intronic | 6 eQTL hits |  |  |  |  |  |  |
| rs2256977 | A/G | 2 | 201208428 | AOX1 | PRO/ANTIOXI | Intronic | 5 eQTL hits |  |  |  |  |  |  |
| rs170548 | A/C | 11 | 107740046 | ATM | REPAIR | Intronic | 7 eQTL hits |  |  |  |  |  |  |
| rs162557 | A/G | 2 | 38158955 | CYP1B1 | PRO/ANTIOXI | Intronic | - |  |  |  |  |  |  |
| rs3212961 | A/C | 19 | 50614163 | ERCC1 | REPAIR | Intronic | 22 eQTL hits |  |  |  |  |  |  |
| rs762562 | A/G | 19 | 50604183 | ERCC1 | REPAIR | Missense | 3 eQTL hits |  |  |  |  |  |  |
| rs3212961 | A/C | 19 | 50614163 | ERCC1 | REPAIR | Intronic | 24 eQTL hits |  |  |  |  |  |  |
| rs3212964 | A/G | 19 | 50612636 | ERCC1 | REPAIR | Intronic | 24 eQTL hits |  |  |  |  |  |  |
| rs50871 | A/C | 19 | 50554355 | ERCC2 | REPAIR | Intronic | 15 eQTL hits |  |  |  |  |  |  |
| rs1635518 | A/G | 1 | 240077197 | EXO1 | REPAIR | 5’-UTR | - |  |  |  |  |  |  |
| rs479744 | C/A | 6 | 109126725 | FOXO3 | INSULIN | Intergenic | 4 eQTL hits |  |  |  |  |  |  |
| rs2267723 | A/G | 7 | 30973467 | GHRHR | INSULIN | Intronic | 6 eQTL hits |  |  |  |  |  |  |
| rs4988505 | C/G | 7 | 30983628 | GHRHR | INSULIN | Intronic | 6 eQTL hits |  |  |  |  |  |  |
| rs512692 | A/T | 3 | 173653536 | GHSR | INSULIN | 5’-UTR | - |  |  |  |  |  |  |
| rs572169 | A/G | 3 | 173648421 | GHSR | INSULIN | Synonimous | 5 eQTL hits |  |  |  |  |  |  |
| rs3756704 | A/G | 5 | 95186050 | GLRX | PRO/ANTIOXI | 5’-UTR | 4 eQTL hits |  |  |  |  |  |  |
| rs2236270 | A/C | 20 | 32986816 | GSS | PRO/ANTIOXI | Intronic | 23 eQTL hits |  |  |  |  |  |  |
| rs7078413 | A/C | 10 | 94280464 | IDE | INSULIN | Intronic | 7 eQTL hits |  |  |  |  |  |  |
| rs12437963 | A/G | 15 | 97314382 | IGF1R | INSULIN | Intronic | 3 eQTL hits |  |  |  |  |  |  |
| rs2252673 | C/G | 19 | 7101418 | INSR | INSULIN | Intronic | 6 eQTL hits |  |  |  |  |  |  |
| rs2283368 | A/G | 13 | 32491270 | KL | INSULIN | Intronic | 4 eQTL hits |  |  |  |  |  |  |
| rs9527026 | A/G | 13 | 32526239 | KL | INSULIN | Synonimous | 4 eQTL hits |  |  |  |  |  |  |
| rs2436514 | A/G | 19 | 5645630 | LONP1 | REPAIR | Intronic | 17 eQTL hits |  |  |  |  |  |  |
| rs512150 | A/T | 11 | 93860853 | MRE11A | REPAIR | Intronic | 30 eQTL hits |  |  |  |  |  |  |
| rs533984 | A/G | 11 | 93838920 | MRE11A | REPAIR | Intronic | 19 eQTL hits |  |  |  |  |  |  |
| rs592068 | A/G | 11 | 93826603 | MRE11A | REPAIR | Intronic | 33 eQTL hits |  |  |  |  |  |  |
| rs10831227 | A/G | 11 | 93795225 | MRE11A | REPAIR | Intronic | 7 eQTL hits |  |  |  |  |  |  |
| rs604845 | A/G | 11 | 93822337 | MRE11A | REPAIR | Intronic | 13 eQTL hits |  |  |  |  |  |  |
| rs12680687 | A/C | 8 | 91020564 | NBN | REPAIR | Intronic | 9 eQTL hits |  |  |  |  |  |  |
| rs2735385 | A/C | 8 | 91018900 | NBN | REPAIR | Intronic | 3 eQTL hits |  |  |  |  |  |  |
| rs449807 | A/T | 9 | 118005570 | PAPPA | INSULIN | Intronic | - |  |  |  |  |  |  |
| rs4837525 | A/G | 9 | 118078379 | PAPPA | INSULIN | Intronic | - |  |  |  |  |  |  |
| rs225119 | A/G | 1 | 7966948 | PARK7 | PRO/ANTIOXI | Intronic | 1 eQTL hit |  |  |  |  |  |  |
| rs1136410 | A/G | 1 | 224621925 | PARP1 | REPAIR | Missense | 3 eQTL hits |  |  |  |  |  |  |
| rs1553850 | A/T | 10 | 120928814 | PRDX3 | PRO/ANTIOXI | 5’-UTR | 11 eQTL hits |  |  |  |  |  |  |
| rs2038526 | A/G | 20 | 48619056 | PTPN1 | INSULIN | Intronic | 2 eQTL hits |  |  |  |  |  |  |
| rs6067484 | A/G | 20 | 48586190 | PTPN1 | INSULIN | Intronic | 1 eQTL hit |  |  |  |  |  |  |
| rs2426164 | A/G | 20 | 48631734 | PTPN1 | INSULIN | Intronic | 2 eQTL hits |  |  |  |  |  |  |
| rs6063534 | A/G | 20 | 48622575 | PTPN1 | INSULIN | Intronic | 2 eQTL hits |  |  |  |  |  |  |
| rs11573709 | A/G | 9 | 109125076 | RAD23B | REPAIR | Intronic | 1 eQTL hit |  |  |  |  |  |  |
| rs11656253 | A/G | 17 | 1708196 | RPA1 | REPAIR | Intronic | 1 eQTL hit |  |  |  |  |  |  |
| rs17292175 | A/G | 17 | 1731525 | RPA1 | REPAIR | Intronic | 2 eQTL hits |  |  |  |  |  |  |
| rs2078486 | A/G | 17 | 7523808 | TP53 | REPAIR | Intronic | 7 eQTL hits |  |  |  |  |  |  |
| rs17202060 | A/G | 12 | 103254976 | TXNRD1 | PRO/ANTIOXI | Intronic | 6 eQTL hits |  |  |  |  |  |  |
| rs11574218 | A/C | 8 | 31057332 | WRN | REPAIR | Intronic | - |  |  |  |  |  |  |
| rs1799782 | G/A | 19 | 48749414 | XRCC1 | REPAIR | Missense | 3 eQTL hits |  |  |  |  |  |  |
| rs3213403 | A/G | 19 | 48738568 | XRCC1 | REPAIR | 3’-UTR | 7 eQTL hits |  |  |  |  |  |  |

Table 4S: Functional prediction of most significant SNPs in SNP pairs found associated with longevity, by SNPsyn analysis

(top-ranked 22 SNP-SNP interactions reported in Table 1) and/or MDR (as reported in Table 2). dbSNP functional annotation

and information about eQTL evidences retrieved by HaploReg database were reported, for estimating a possible functional

impact of the SNPs (<http://archive.broadinstitute.org/mammals/haploreg/haploreg.php>.)
